# *Saccharina* genomes provide novel insight into kelp biology

**DOI:** 10.1038/ncomms7986

**Published:** 2015-04-24

**Authors:** Naihao Ye, Xiaowen Zhang, Miao Miao, Xiao Fan, Yi Zheng, Dong Xu, Jinfeng Wang, Lin Zhou, Dongsheng Wang, Yuan Gao, Yitao Wang, Wenyu Shi, Peifeng Ji, Demao Li, Zheng Guan, Changwei Shao, Zhimeng Zhuang, Zhengquan Gao, Ji Qi, Fangqing Zhao

**Affiliations:** 1 Yellow Sea Fisheries Research Institute, Chinese Academy of Fishery Sciences, Qingdao 266071, China; 2 Computational Genomics Lab, Beijing Institutes of Life Science, Chinese Academy of Sciences, Beijing 100101, China; 3 College of Life Sciences, University of Chinese Academy of Sciences, Beijing 100049, China; 4 Tianjin Key Laboratory for Industrial Biosystems and Bioprocessing Engineering, Tianjin Institute of Industrial Biotechnology, Chinese Academy of Sciences, Tianjin 300308, China; 5 School of Life Sciences, Shandong University of Technology, Zibo 255049, China; 6 State Key Laboratory of Genetic Engineering and Collaborative Innovation Center for Genetics and Development, Institute of Plant Biology, School of Life Sciences, Fudan University, Shanghai 200433, China

## Abstract

Seaweeds are essential for marine ecosystems and have immense economic value. Here we present a comprehensive analysis of the draft genome of *Saccharina japonica*, one of the most economically important seaweeds. The 537-Mb assembled genomic sequence covered 98.5% of the estimated genome, and 18,733 protein-coding genes are predicted and annotated. Gene families related to cell wall synthesis, halogen concentration, development and defence systems were expanded. Functional diversification of the mannuronan C-5-epimerase and haloperoxidase gene families provides insight into the evolutionary adaptation of polysaccharide biosynthesis and iodine antioxidation. Additional sequencing of seven cultivars and nine wild individuals reveal that the genetic diversity within wild populations is greater than among cultivars. All of the cultivars are descendants of a wild *S. japonica* accession showing limited admixture with *S. longissima*. This study represents an important advance toward improving yields and economic traits in *Saccharina* and provides an invaluable resource for plant genome studies.

## Abstract (web summary)

Kelps are ecologically and economically important seaweeds. Here the authors sequence the genome of *Saccharina japonica* to gain insights into the evolutionary adaptation of polysaccharide biosynthesis, iodine concentration and antioxidation mechanisms and the population genetics of kelp domestication.

## Graphical abstract

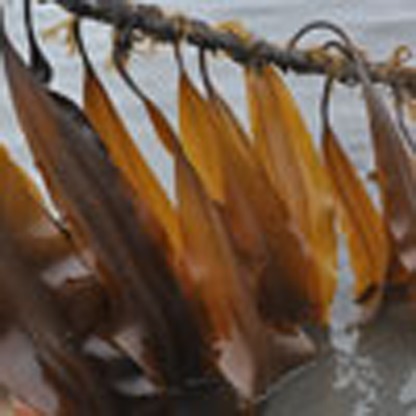

Brown macroalgae (kelps) belong to the phylum Stramenopiles, a phylogenetic lineage that is distantly related to terrestrial plants and animals^1^. These macroalgae exhibit differentiated tissues during development, making them distinct from unicellular stramenopiles. The extensive submarine kelp forests are the largest biogenic structures within benthic marine communities, occupying 70% of the total biomass in cold and temperate marine systems^2^. Specifically, Laminariales kelp species are essential for ecosystems and are economically important as a marine crop. These kelps are cultivated in East Asia and harvested from natural populations in Europe and North America to produce of alginate, which is used in a wide variety of pharmaceuticals, foods and industrial applications^3^. These kelps may also provide an important component for the future renewable energy mix^4^. Furthermore, Laminariales are the largest accumulators of iodine, contributing tremendously to the biogeochemical iodine cycle, and thus have a significant impact on atmospheric chemistry^5^. In particular, *S. japonica*, one of the most common Laminariales alga along the northwest coasts of the Pacific Ocean^6^, is becoming the most economically important seaweed in the sea farming cultivation industry. On the basis of artificial seedling-rearing techniques, *S. japonica* sea farming has evolved rapidly to make it the most common seaweed in the world^7^, with most of this seaweed used as food and raw industrial materials. The output of *S. japonica* reached 7.9 million tons (dry weight) and had a market value of more than US $1.3 billion in 2012 (ref. 8).

Despite the ecological, economic and evolutionary importance of kelps, we currently have a very limited knowledge of their genetic architecture and metabolism, including their iodine concentration system and alginate-producing pathway, a limitation that hinders both genetic research and mariculture practices. Furthermore, years of interspecific hybridization and biomass yield-targeted artificial selection have not only degeneratted the economic characteristics of these kelps but have also narrowed their genetic variation. The recently sequenced small filamentous model brown alga *Ectocarpus siliculosus*^9^, a close relative of kelp species^10^, greatly facilitated the functional and evolutionary investigation of *S. japonica* in this study. Here we report a draft genome sequence of the female gametophytes of the artificially cultivated *S. japonica* strain Ja and the resequencing of seven wild populations and nine representative cultivars of *Saccharina* species. Comparative genomic analyses of these data provide novel insight into the evolutionary adaptation and the functional diversification of the polysaccharide biosynthesis and iodine concentration mechanisms of *S. japonica*. The *Saccharina* genomic sequence that was obtained represents an important advance toward securing bioproducts and biofuels from macroalgae and provides an invaluable resource for plant gene and genome evolution studies.

## Results

### Genome sequencing and assembly

Genomic DNA was extracted from filamentous female gametophytes of *S. japonica* strain Ja cultured in Qingdao, China. The DNA sequencing reads were obtained using both Roche and Illumina technologies and were assembled after filtering out the low-quality and duplicated reads. A total of 84 Gb of high-quality Illumina reads and 10 Gb of PacBio long reads (Supplementary Table 1) were generated, representing an ∼178 × coverage of the *S. japonica* genome, with an estimated size of 545 Mb based on kmer depth distribution analyses and flow cytometry (Supplementary Figs 1 and 2). Approximately 98.5% (537 Mb) of the genome was *de novo* assembled, consisting of 13,327scaffolds (≥500 bp) with a scaffold N50 length of 252,007 bp (longest, 1.47 Mb) and a contig N50 length of 58,867 bp (Supplementary Table 2).

### Genome annotation and gene prediction

**Figure 1:**
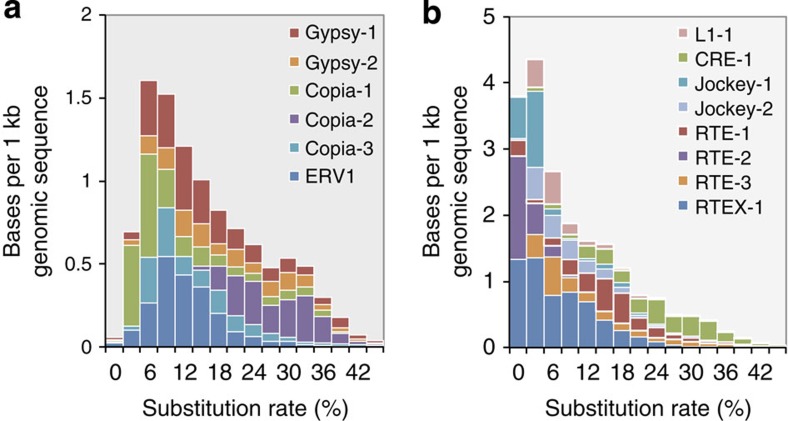
The repetitive elements in *S. japonica*. (**a**) Age distribution of the LTR elements in *S. japonica*. (**b**) Age distribution of the LINE elements in *S. japonica*. The average number of substitutions per site for each fragmented repeat was estimated using the one-parameter Jukes–Cantor model. Insertions and deletions were excluded from the substitution rate calculation. The per cent substitution from the consensus roughly correlates with the age of the repetitive elements. LINE, long interspersed elements.

By combining homology-based and *de novo* approaches, we identified ∼40% of repetitive elements in the assembled *S. japonica* genome, which is nearly twice as many as in *E. siliculosus* (22.7%) (Supplementary Fig. 3). Among these repeats, 63.1% could be classified into known repeat families, with long-terminal repeats (LTRs) constituting the most abundant repeat family, representing 21.1% of the repetitive sequences. Long interspersed elements were the second largest family found in *S. japonica* and could be subdivided into Jockey, RTE, L1 and CRE elements. Compared with the LTRs (for example, Copia and Gypsy), the L1 and the CRE elements and the RTE-2 and the RTEX-1 retrotransposons identified in *S. japonica* shared a high sequence similarity (>95%), indicating that they were recently inserted into the *S. japonica* genome and are likely to be active in the amplification (Fig. 1). Previous studies have demonstrated that RTEs exhibit a mosaic distribution throughout the Animalia kingdom and tend to spread via horizontal gene transfer (HGT)^11^,^12^. A comprehensive search of the RTE RT domains in published algal genomes revealed that *E. siliculosus* possesses a significant fraction of RTE elements (1.6%) that share high sequence similarity with those found in *S. japonica*, whereas the RTEs identified in two green algal genomes (*Volvox carteri* and *Chlamydomonas reinhardtii*) were relatively divergent. A phylogenetic analysis of the RTE elements showed that they were not consistent with the species tree, indicating the lateral gene transfer of the RTE elements in these organisms. (Supplementary Fig. 4).

To more accurately annotate the *S. japonica* genome, we performed deep transcriptome sequencing of both female gametophytes and sporophytes, which generated 11.3 Gb of RNA sequencing data. By combining homologue-based, *ab initio* and transcriptome-based approaches, we predicted 18,733 protein-coding genes (gene models) in the *S. japonica* genome, which is greater than the number of genes predicted in *E. siliculosus*^7^ (16,256). The average gene size (exons and introns) was 9,587 bp, with 6.54 exons per gene and an average intron size of 1,057 bp, which is significantly larger than in *E. siliculosus* (average intron size, 703 bp). More than 90.7% of the predicted coding sequences were supported by transcriptome sequencing data (≥5 reads), indicating the high accuracy of the gene predictions in the sequenced *S. japonica* genome (Supplementary Fig. 5). To independently evaluate the genome completeness, we found that 96.5% of *de novo* assembled transcripts (25,010 out of 25,914) could be aligned to the assembled genome. Among the annotated genes, 86.1% of the encoded proteins had homologues in the NCBI non-redundant protein database, with 70.1% of the putative proteins showing best-hit matches to *E. siliculosus*. Previous studies have revealed that *E. siliculosus* possesses an integrated virus (EsV-1) in its genome^9^,^13^. In this study, we did not find any homologous genes of EsV-1 in the *Saccharina* genome. However, using PFAM domain searches, we identified 121 putative proteins in *S. japonica* containing the FNIP repeat domain (PF05725), which is ∼22 residues long and has previously only been found in *Dictyostelium* and a few double-stranded DNA viruses. Similar FNIP repeat domains have also found in the *E. siliculosus* genome. A phylogenetic analysis based on the FNIP repeat domain revealed that the closest relatives of these FNIP sequences came from a giant virus (Supplementary Figs 3B and 6) that infects the marine zooplankton *Cafeteria roenbergensis*^13^, suggesting an ancient association of brown algae with viruses.

### Comparative genomics

**Figure 2:**
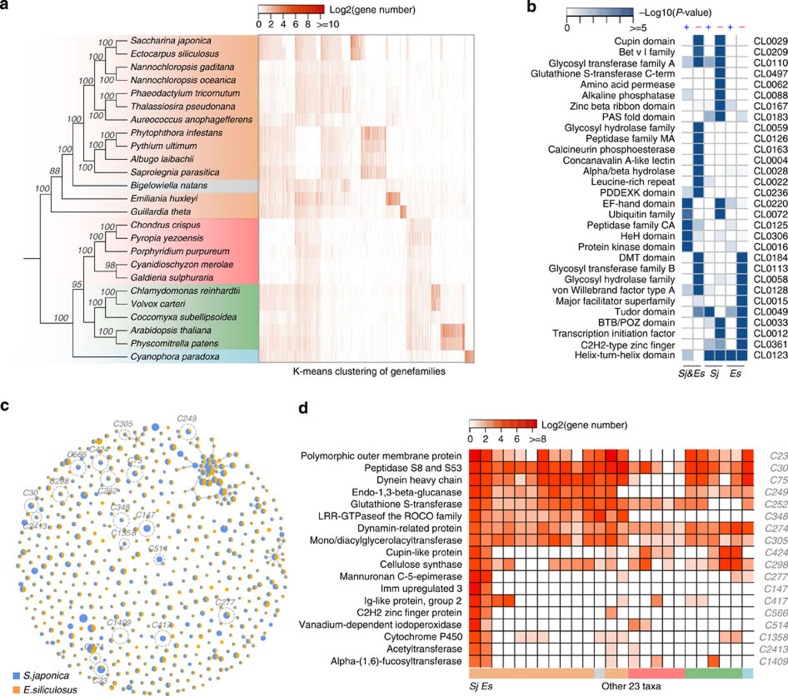
The comparative genomics of *S. japonica* and 24 other algae and plant genomes. (**a**) The phylogenetic tree was constructed based on the concatenated sequences of 41 single-copy genes in all of these genomes after the removal of species-specific gene duplications using maximum-likelihood methods. The coloured squares denote Chromalveolata (brown), Rhodophyta (red), Viridiplantae (green), Rhizaria (grey) and Glaucophyta (blue). A K-means clustering of the families based on the gene abundance in each species is shown in the right panel; each column represents a family and each row represents one species. The clustering is sorted according to the order of the left tree. (**b**) The domain-based annotation of the gained/lost families in *S. japonica* or *E. siliculosus* or their latest common ancestor. Fisher's exact test of the domain enrichment of the families was compared against the total number of annotated domain in both of the genomes. (**c**) A comparison of the gene abundances between *S. japonica* and *E. siliculosus* in their shared families. The pie charts at each node represent the ratio of *S. japonica* genes (blue) to *E. siliculosus* genes (yellow) in each gene family. (**d**) The copy number differences of the genes in each family within which *S. japonica* (the first column) underwent more gene amplifications than *E. siliculosus* (the second column).

To investigate the gene content of *S. japonica*, phylogenomic analyses of 25 genomes from Chromalveolata, Rhizaria, Glaucophyta, Rhodophyta, Chlorophyta and higher plants were performed. First, 41 orthologues of single-copy or species-specific gene duplications were identified (Supplementary Data 1; Supplementary Fig. 7A) for a phylogenetic tree reconstruction using concatenated super genes after the removal of redundancy to avoid the effects of loss of paralogues. As shown in Fig. 2a, the phylogenetic tree divided the 23 organisms into three main phyla, Chromalveolata, Rhodophyta and Viridiplantae. A total of 19,410 gene families were then predicted from the 404,604 genes of *S. japonica* and the other 24 genomes (Supplementary Data 2). Among these gene families, a Dollo parsimony analysis based on the genomes of the seven heterokontic algae revealed that many large-scale amplification events in different clades: 400 gene families were gained in the ancestor of diatoms, 451 in *Nannochloropsis*, and strikingly, 1,240 families were gained in the common ancestor of *S. japonica* and *E. siliculosus* (Supplementary Data 3; Supplementary Fig. 7B). Consistently, these amplification events were also observed in a K-means clustering of the families based on the gene abundance of each species (Fig. 2a, right panel; Supplementary Fig. 8). In addition, some clades were found to have lost many gene families, namely, 499 families were lost in the diatom clade and 813 in *Nannochloropsis*. The loss of so many gene families greatly reduced the genome sizes of these species. Conversely, because more gene families were gained rather than lost, brown algae experienced a large genome expansion during evolution.

After the divergence of Phaeophyceae and Eustigmatophyceae, *S. japonica* and *E. siliculosus* together exhibited a significant increase in their gene family number (1,240 gained versus 309 lost). Because of insufficient evidence of protein similarities to other algae, most of these newly gained families in Phaeophyceae were classified as proteins with unknown function. It should be noted that 102 annotated gained families were found to be significantly enriched (*P*<10^−5^) in the protein kinase or HeH or peptidase CA super family domains (or clans; Fig. 2b). By contrast, the genes from the 309 families that were lost in the two species showed a relatively good annotation and were enriched in the clans of the cupin domain (4.5% of 309 families), the alpha/beta hydrolase (2.9%), the glycosyl hydrolase family (1.9%) and a wide range of other clans (Fig. 2b).

*S. japonica* and *E. siliculosus* share 4,309 gene families, which comprise 17,379 genes in *S. japonica* and 14,136 genes in *E. siliculosus*, covering 92.8% and 85.5% of the gene content of each genome, respectively. The higher number and content of these gene families in *S. japonica* leads to a hypothesis that gene family expansion events occurred more frequently in *S. japonica* than in *E. siliculosus*. Among the shared gene families, 2,267 of them only had one copy in each genome, whereas 863 (20%) families were found to have more gene copies in *S. japonica* (9,562 of the total genes) than in *E. siliculosus* (4,666), but only 652 families were identified to be the opposite (3,753 for *S. japonica* versus 5,406 genes for *E. siliculosus*). These results indicate that the majority of the amplified genes found in *S. japonica* resulted from recent duplication events (with an average synonymous substitution rate of 0.42; Supplementary Fig. 9), because the mean similarity among them (79%) was higher than the similarity observed between the two species (74%).

*S. japonica* and *E. siliculosus* also accumulated hundreds of gene families independently through gain/loss events after their diversification, which further contributed to the difference in their gene content, with 527 genes gained in *S. japonica* and 629 gained in *E. siliculosus*. A total of 574 large families (Fig. 2c) with 10 or more genes from the two Phaeophyceae genomes were selected for further consideration. Compared with *E. siliculosus*, *S. japonica* has significant gene expansion in 58 families (Fisher exact test, corrected *P*-value<0.05), including those families involved in iodine concentration and the biogenesis and remodelling of cell wall polysaccharides, for example, vanadium-dependent haloperoxidases (vHPOs) that catalyse the oxidation of halides^14^, cellulose synthase (GT2 family) that catalyses the terminal step of cellulose biosynthesis, mannuronan C-5-epimerases (MC5Es) that epimerize D-mannuronate residues into L-guluronate for alginate biosynthesis and alpha-(1,6)-fucosyltransferase (GT23 family) that polymerize GDP-fucose into the elongated fucan chain^15^ (Fig. 2d). Other examples come from families encoding the endo-1,3-beta-glucanase (GH81 family), leucine-rich GTPase and Imm upregulated gene families, which may be related to the development and defence systems in brown algae (Fig. 2d). Endo-1,3-beta-glucanase hydrolyses the glycoside linkages of laminarin, the major storage polysaccharide in *S. japonica*, into oligosaccharides that respond to tissue damage and provide protection against pathogens by triggering defence responses such as an initial oxidative burst^16^. They are also thought to play important roles in diverse physiological and developmental processes in plants such as microsporogenesis, fertilization, seed germination and somatic embryogenesis^17^. The leucine-rich GTPases of the ROCO family, which are excellent candidates for recognition/transduction events linked to immunity in *Ectocarpus*^18^, are another potential family involved in defence and development. The Imm upregulated genes are brown algae-specific gene families that were first discovered in *E. siliculosus*, and are related to the development of the sporophyte and gametophyte generations^19^. Imm upregulated 3 was found to be significantly upregulated in the gametophyte generation compared with the sporophyte generation^16^. It was also found to be a female-biased gene in the brown alga *Fucus vesiculosus*^20^. In addition, this gene has weak similarity to BIP2, a gene that appears to be specifically associated with the acquisition of a three-dimensional architecture in *Physcomitrella*^21^. In addition to these, several super families with diverse functions, including Cupin-like proteins, Ig-like proteins, C2H2 zinc-fingrer proteins and cytochrome P450 were also expanded in *S. japonica*.

### Expansion of vHPO genes is associated with functional diversification

**Figure 3:**
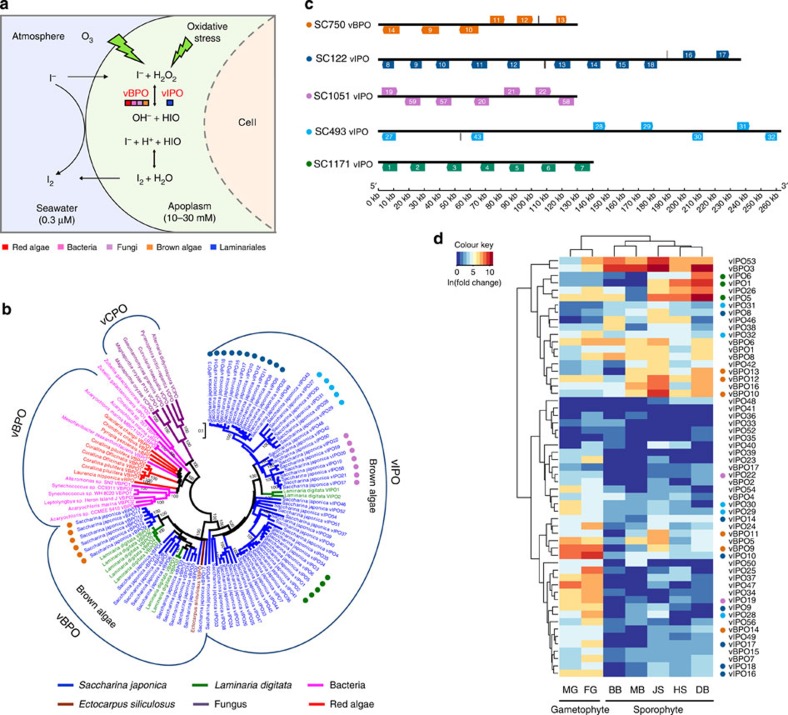
The vBPO and vIPO genes involved in halogen metabolism in *S. japonica*. (**a**) A schematic representation of the iodine uptake and release in Laminariales in response to oxidative stress involving vBPOs and vIPOs. The coloured squares denote the homologous genes found in red algae (red), bacteria (pink), fungi (purple), brown algae (orange) and Laminariales (blue). The vIPOs are only found in Laminariales species. (**b**) The genomic organization of one vBPO and four vIPO gene clusters, which are arranged in tandem order. The numbers in the different coloured boxes represents the vBPO/vIPO gene names. The hypothetical proteins are shown in grey boxes. SC, scaffold. (**c**) The phylogenetic analysis of the vHPO gene families. Five recent tandem duplicated gene clusters are indicated with circles of different colours. (**d**) The heat map of the expression profiles of the vBPO and vIPO genes in gametophytes, juvenile sporophytes and different tissues of adult sporophytes of *S. japonica*. Recent tandemly duplicated vHPO genes, indicated with coloured circles, show obvious expression diversification. BB, basal blade; DB, distal blade; FG, female gametophytes; HS, holdfast and stipe; JS, juvenile sporophyte; MB, middle blade; MG, male gametophytes.

Brown macroalgal species are the most well-known effective iodine accumulators among all living organisms and are major contributors to the global biogeochemical iodine cycle, displaying an average iodine content of 1% (up to 5%) of their dry weight, representing ∼30,000 times the concentration of this element in seawater^22^. In addition, these species are the only known organisms to use inorganic iodide as an extracellular antioxidant in a living system^23^. Most iodine compounds are chelated by apoplastic macromolecules and accumulate in the apoplast of the cortical cell layer, which protects the thallus surface from both aqueous and gaseous oxidants^23^. However, the mechanisms of iodine concentration and antioxidation are not well known and are presumably linked to the presence of particular vHPOs. In algae, vHPOs are a particular class of peroxidases that catalyse the oxidation of halides in the presence of hydrogen peroxide, leading to the halogenation of various organic substrates^14^. In *Laminaria*, the identified vHPOs comprise two large multigenic families encoding vanadium-dependent bromoperoxidases (vBPOs) and iodoperoxidases (vIPOs)^24^. Previously, vIPOs had mostly been found in Laminariaceae species, and they are characterized by the novel biochemical function of showing strict specificity for iodide oxidation (Fig. 3a).

In the *S. japonica* genome, 17 vBPO and 59 vIPO genes were identified and annotated, and a phylogenetic analysis revealed that all of the vHPO genes form a monophyletic group sharing a common ancestor with the vCPO (vanadium-dependent chloroperoxidase) genes of fungi, after which they evolved independently in red and brown algae (Fig. 3b; Supplementary Table 3), which is consistent with a previous study that found that vIPOs and vBPOs were paralogues resulting from an ancestral gene duplication^24^. Recently, two bacterial vIPO genes were found in the flavobacterium *Zobellia galactanivorans*, a marine bacterium associated with macroalgae^25^. A phylogenetic analysis showed that these two bacterial vIPO genes evolved independently from eukaryotic algal vHPO (Fig. 3b). A pairwise similarity comparison of vHPOs revealed at least five blocks of conserved gene clusters that are expected to have derived from recent tandem duplication events (Fig. 3b,c; Supplementary Fig. 10). The transcriptional regulation of vHPO has been shown to be efficient for switching to the specialized iodine metabolism related to antioxidative capacities^26^. The expression of 59 vHPO genes was determined in *S. japonica* gametophytes, juvenile sporophytes and in different tissues of adult sporophytes, including the holdfast, stipe, basal blades, middle blades and distal blades (Supplementary Table 4). Notably, the vHPO genes identified in *S. japonica* showed diverse expression patterns in different tissues and during different developmental stages. In particular, vHPO gene expression was observed to be significantly upregulated in the gametophytes, which is the stage that is most vulnerable to external stress in the entire life circle of *S. japonica*. Among the sporophyte samples, the greatest number (54) and the amount of vHPOs were expressed in the juvenile sporophytes, followed by the distal blades (53). These results agree well with the largest quantity of iodine elements being found in the juvenile sporophytes and distal blades in *L. digitata*^23^ and *S. japonica* (Supplementary Fig. 11). During the cultivation of kelps, the juvenile sporophytes and distal blades are more sensitive to environmental stresses and are more readily infected by pathogens^27^. Notably, the expression of vIPOs is more specific than the expression of vBPOs, with only 66.7% of the tested vIPOs being expressed in all samples, compared with 82.3% of vBPOs. One possible explanation for this pattern is that recently tandem duplicated vIPO genes showing high sequence identities and distinct expression patterns in gametophyte and sporophyte, exhibit functional diversification in *S. japonica* (Fig. 3d).

### Carbohydrate pathways in *S. japonica*

**Figure 4:**
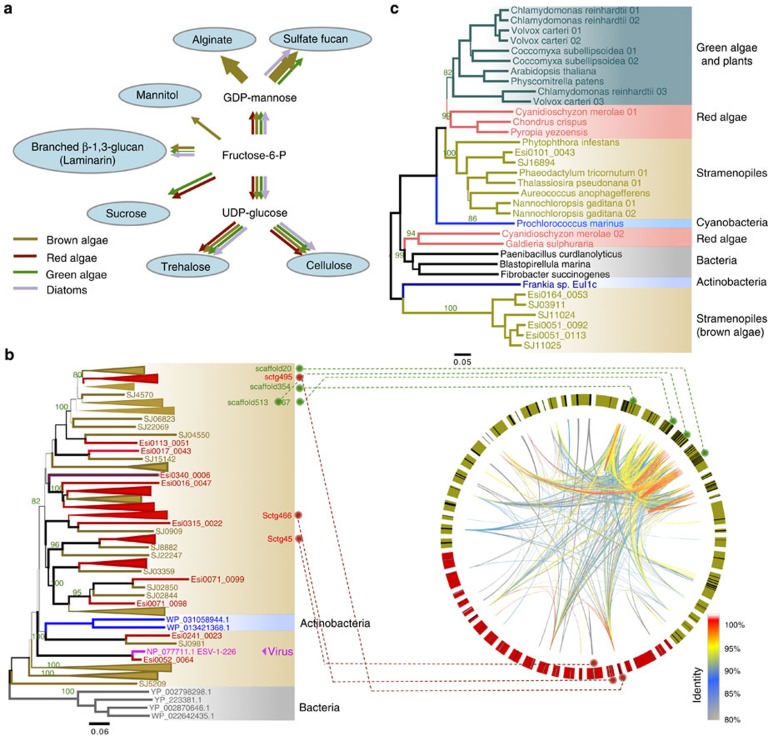
Polysaccharide biosynthesis and metabolism in *S. japonica*. (**a**) A comparison of the carbohydrate metabolic pathways among algal genomes. The width of the arrows represents the number of genes involved in the metabolic pathway. (**b**) A phylogenetic analysis and Circos plot of MC5E genes in *S. japonica* and *E. siliculosus*. The majority of the phylogenetically clustered MC5E genes present in *S. japonica* are in the assembled scaffolds in a tandem order. The brown nodes and red nodes in the tree denote the MC5E genes derived from *S. japonica* and *E. siliculosus*, respectively. The abbreviated words begin with ‘Esi' and ‘SJ' in the tree stand for the protein ids for *E. siliculosus* and *S. japonica*, respectively. Actinobacteria, Bacteria and Virus taxon names were represented by the NCBI protein accession numbers. The *S. japonica* and *E. siliculosus* scaffolds in the Circos plot are shown in brown and red, respectively. Green and red dashed lines represent the locations of MC5E-enriched scaffolds in *S. japonica* and *E. siliculosus*, respectively. The inner curved lines in the Circos plot indicate the sequence identity between a pair of MC5E genes. (**c**) The phylogenetic analysis of GMD, a key enzyme in the alginate biosynthesis pathway.

Compared with land plants and green or red algae, brown algae exhibit unique modes of carbon storage and cell wall metabolism^9^,^15^,^28^,^29^. Cellulose and trehalose are common in both algae and land plants. However, a striking difference in brown algae is that they utilize mannitol and laminarian for carbon storage and possess alginates and sulfated fucans as cell wall polysaccharides. By reconstructing the carbohydrate metabolism pathways in *S. japonica* and 14 other algal genomes, we found that *S. japonica* harbours the same carbohydrate pathways as *E. siliculosus* and is distinct from *Aureococcus* and *Nannochloropsis* in the alginate pathway and from diatoms in the mannitol and alginate pathways. The starch and sucrose pathways are absent in all four of the stramenopiles species (Fig. 4a; Supplementary Fig. 12).

Notably, in *S. japonica*, gene expansion and duplication events were observed at particular reaction nodes of the alginate and sulfated fucan pathways. Alginate is the major matrix component of brown algal cell walls, providing an increased rigidity to the stipe and holdfasts as well as flexibility to the blades^3^. The different matrix and physicochemical properties of alginate depend on the final step of epimerization, which is a reaction catalysed by MC5Es^27^. *S. japonica* was found to exhibit 105 MC5E genes, whereas only 28 were identified in *E. siliculosus*. In addition, 43 of the MC5E genes present in *S. japonica* shared high sequence identities (>85%) and were clustered on seven scaffolds (Fig. 4b), indicating recent tandem duplication events. The expansion of MC5Es in *S. japonica* may account for the species' extraordinarily high alginate content (up to 45% of the dry weight)^28^ and high bending flexibility.

A phylogenetic analysis showed that the MC5E genes of brown algae were similar to the MC5E genes in bacteria (Fig. 4b), indicating that these genes may have undergone a non-canonical evolutionary history and then expanded into several subfamilies through multiple duplication events. A prior study in *E. siliculosus* hypothesized that this evolutionary history involved HGT in (Supplementary Fig. 13)^27^. A similar phenomenon was observed for another alginate-specific enzyme, GDP-mannose dehydrogenase. One GDP-mannose dehydrogenase gene was found to be conserved in all Chromalveolata species, whereas the other three clustered in a distinct clade that comprises only bacterial species and *E. siliculosus*, indicating that these three enzymes were acquired via ancient HGT from bacteria before the last common ancestor of brown algae (Fig. 4c; Supplementary Table 5). In addition, the mannitol synthesis pathway, which is catalysed by mannitol-1P dehydrogenase and mannitol-1-phosphatase, is not universally present in all stramenopiles. Both of these genes are absent from diatoms and oomycetes but were surprisingly found in the Haptophyceae alga *Emiliania*. Phylogenetic analyses suggest that the mannitol pathway may have been acquired by the common ancestor of brown algae and Haptophyceae (Supplementary Fig. 14). No Actinobacteria species harbours all of the brown algal MC5E, mannitol-1P dehydrogenase and mannitol-1-phosphatase homologues, indicating that the abundant, complex polysaccharide pathways observed in brown algae were likely acquired through multiple independent HGT or other non-canonical events (Fig. 4b).

### Population genomics

**Figure 5:**
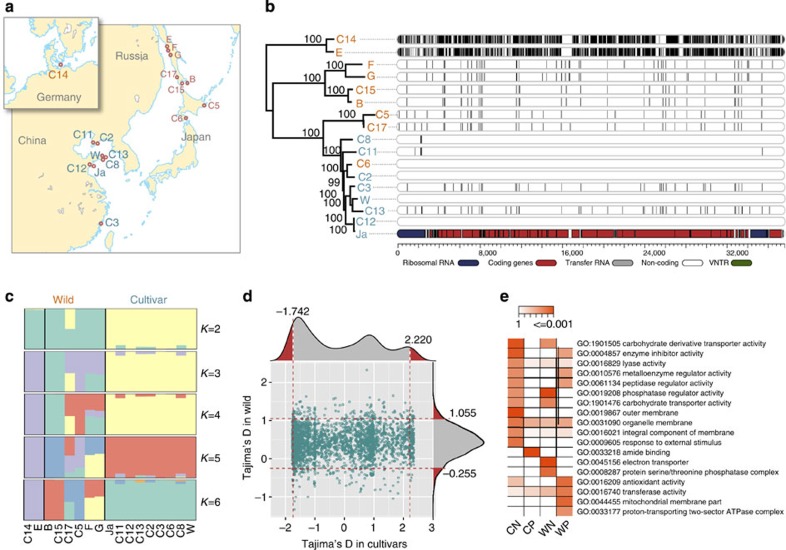
The population genomics of cultivated and wild *S. japonica* populations. (**a**) The geographic locations of the cultivated and wild *S. japonica* samples used in this study. (**b**) The neighbour-joining tree of the genetic distances calculated using the genome-wide SNVs among 17 *S. japonica* individuals. The mitochondrial diversity covering SNVs is indicated with vertical lines in the seven cultivated and nine wild samples relative to the *S. japonica* JA reference sequence. (**c**) The population structure of 17 *S. japonica* individuals determined using the STRUCTURE program. Each individual is represented by a vertical bar, and each colour represents one population. (**d**) The distribution of the Tajima's D values in both the wild and cultivated populations. The data points to the left and right of the vertical dashed lines (*x*=−1.742 and *x*=2.220), which correspond to the 5% left and right tails of the empirical Tajima's D value distribution, respectively, are denoted as CN (cultivated negative) and CP (cultivated positive), respectively. The data points below and above the lower and upper horizontal dashed lines (*y*=−0.255 and *y*=1.055), which correspond to the 5% lower and upper tails of the empirical Tajima's D value distribution, respectively, are denoted as WN (wild negative) and WP (wild positive), respectively. (**e**) The gene ontology-enrichment analysis of the genes in the selected CN, CP, WN and WP regions.

To better understand the genetic diversity of the *Saccharina* population, we resequenced the entire genomes of seven cultivated individuals from China and nine wild individuals collected from Japan, Russia and Germany (Fig. 5a). We first compared the number of mitochondrial differences in these 17 *Saccharina* individuals and found that the cultivated populations exhibited relatively low levels of genetic diversity compared with the wild populations. By contrast, *S. latissima* (C14) and a wild isolate (‘E'; as shown in Fig. 5b) possessed a much higher genetic diversity than the other 15 *Saccharina* individuals, showing 1,162 and 1,214 polymorphic loci in their mitochondrial genomes, respectively. *S. latissima* is a close relative of *S. japonica*, and the former is considered a native European species (Fig. 5b). However, its close relative *S*. sp. E is also present along Russia's Far Eastern coast. After aligning the reads to the reference Ja genome, we identified an average of 0.94 M single-nucleotide variations (SNVs) and 96-K-small InDels in the cultivars and an average of 2.27 M SNVs and 274-K-small InDels in the wild populations. In addition, considering the large genetic distance among *S. latissima* and *S*. sp. E and the reference Ja, we first built draft genome assemblies for *S. latissima* and *S*. sp. E and then employed NUMMER^25^ to align the assembled contigs and to call the SNVs. Phylogenetic analyses based on genome-wide SNVs further confirmed that the diversity between any pair of the collected wild individuals was greater than the diversity among all of the cultivars and that these cultivated individuals shared the same ancestor with the wild individual (C6; Fig. 5b), indicating a restricted germplasm base and a very low genetic diversity within the principal *Saccharina* cultivars. A log likelihood STRUCTURE analysis^15^ revealed that some crossbreeds (C17, C11 and C2) were likely derived from the hybridization of *S. japonica* and *S. longissima* (C5). The number of groups identified within the wild *Saccharina* individuals increased with an increasing number of K, with up to four groups at *K*=5, which was consistent with the phylogenetic analysis (Fig. 5b,c).

To detect the genome-wide signatures of artificial selection in cultivated *Saccharina*, we used a sliding window strategy to estimate the theta-pi and theta-w values from both the cultivated and wild populations and to perform Tajima's D test (Fig. 5d; Supplementary Data 4–7). The regions showing Tajima's D values that were lower or higher than 5% of all of the bins were considered to be candidate regions. We identified 122 regions exhibiting strong signals of a selective sweep in the cultivars, which encompassed 828 genes. A gene ontology-enrichment analysis revealed that the majority of the genes affected by artificial selection were related to carbohydrate transporters, enzyme inhibitors and responses to external stimuli (Fig. 5e). Average yield is one of the most important economic and selective criteria used in *S. japonica* cultivation and seedling selection. The efficiency and regulation mechanisms of carbon fixation and carbohydrate metabolism are important for algal growth and body size. Six genes with known functions exhibiting strong selective signals were significantly over-represented in the cultivated samples. For example, fructose-1,6-bisphosphate aldolase, which catalyses the reversible aldol cleavage of fructose-1,6-bisphosphate into DHAP and glyceraldehyde-3-phosphate, functions in glycolysis or gluconeogenesis and in the Calvin cycle^30^. Traditionally, FbA was assumed to only exist in the genomes of bacteria and fungi, but it has now been found in some species of Chromista and Chromalveolata, including the brown algae *E. siliculosus* and *S. japonica*, giving rise to the possibility that these genes were independently obtained from fungi through ancient HGT events.

A population-level analysis further revealed selection in the wild samples. A total of 566 genes embedded in selected regions presented significantly elevated Tajima's D values. Compared with the cultivars, three transmembrane receptor kinase genes in the wild samples showed strong selective signals that may be related to environmental adaptation and morphological changes. Wild brown algae exhibit a particularly high flexible morphology, and the physical properties of the marine environment are partly responsible for the algal shape. Compared with cultivated kelps on artificial floating rafts with suitable and consistent environments (for example, sufficient water depth), wild kelps show greater morphological plasticity in response to harsh intertidal environments^31^. In land plants, several types of transmembrane receptor kinases transmit information regarding mechanical forces from the cell wall to cytoplasmic effectors and are responsible for the development and morphogenesis^32^,^33^.

## Discussion

Brown algae are valuable economic and ecological resources and are an important evolutionary lineage because of the development of specialized tissues and organs outside of plants and metazoans, which makes their genomes exciting resources for comparative investigations in this field. The S. japonica genome is the first sequenced reference genome from kelps and the second genome from brown algae. When compared with filamentous *E. siliculosus*, *S. japonica* shows several unique characteristics, such as more complex differentiation, large blades, a higher polysaccharide content and striking iodine concentrating abilities. In this study, some of the mechanisms underlying these characteristics were identified using comparative genomics. *S. japonica* can grow up to dozens of metres with large blades, and its cell wall polysaccharide content is much higher than *Ectocarpus*. We speculated that the expansion of the cellulose synthase, mannuronan C-5-epimerase and alpha-(1,6)-fucosyltransferase gene families may be responsible for the formation of the extensible cell walls of *S. japonica* that support its large blade structure^34^. Laminarin is not only the major storage polysaccharide in *S. japonica*, but it also play important roles in the defence system, in which it can be hydrolysed into oligosaccharides by endo-1,3-beta-glucanase. In addition, laminarin and its derived oligosaccharides can even activate the defence response and protect against pathogens in terrestrial plants^16^. Iodide was found to be concentrated by vHPO and was shown to bind to the cell wall polysaccharide components^5^,^26^, providing extracellular antioxidants on the blade surfaces of *S. japonica*. The expansion of the endo-1,3-beta-glucanase and vHPO gene families in *S. japonica* may provide it with more complex defence systems than *Ectocarpus*. Moreover, tissue differentiation was more complex in *S. japonica* than in *Ectocarpus* with respect to such events as the emergence of the holdfast, stipe and blades. In addition to several other expanded gene families, including Cupin-like protein^35^, C2H2 zinc-finger protein^36^ and LRR-GTPase of the ROCO family^37^ (Fig. 2d), which have been shown to have key roles in development in both animals and green plant lineages, the newly identified development related imm upregulated 3 gene family may represent a new developmental mechanism in brown algae. Combining all of the above evidence further indicates that brown algae evolved developmental complexity independently from higher plants and animals.

This work also presents the first comprehensive resequencing data for wild and cultivated *Saccharina* individuals, providing the basic materials for population genetic studies. The *Saccharina* cultivars that are currently farmed in China were mainly bred from the descendants of wild *S. japonica* that were introduced from northern Japan. According to our results, only limited crossbreeds were derived from the hybridization of *S. japonica* and *L. longissima*. Therefore, the restricted germplasm base and continuous selfing of these kelps have resulted in very low genetic diversity within *Saccharina* cultivars. The obtained genomic information on wild *Saccharina* species can not only be used to increase the genetic diversity through hybridization, but it can also provide a large source of candidate genes for further functional studies aimed at improving quality and yield. There are other important issues that remain for the further exploitation of these kelps, such as the many biological features that are unique to *Saccharina*, that need to be studied in detail at the molecular level. In addition, the identification of SNVs in representative wild and cultivated individuals will provide opportunities for marker-assisted breeding.

## Methods

### Genome sequencing and assembly

DNA libraries harbouring 180-bp, 300-bp, 500-bp, 800-bp, 3-kb and 5-kb inserts were subsequently constructed for *S. japonica* Ja and DNA libraries with 300-bp inserts were constructed for the other 16 *Saccharina* individuals. These DNA libraries were paired-end sequenced on an Illumina HiSeq 2000 sequencer. Two-large-insert 454 libraries (8 and 16 kb) were also constructed for *S. japonica* Ja and were sequenced using 454 pyrosequencing. For PacBio library construction, genomic DNA was sheared to 8 kb using an ultrasonicator and was converted into the proprietary SMRTbell library format using an RS DNA Template Preparation Kit. SMRTbell templates were subjected to standard SMRT sequencing on the PacBio RS system according to the manufacturer's protocol.

We used the paired-end reads from the short-insert-size libraries (180, 300, 500 and 800 bp) to assemble the genome into contigs using SOAPdenovo2 (ref. 38) with the multi-kmer option (-K 63 -m 81). We then aligned all of the available sequence data to these contigs and used the mate-pair information on the order of the estimated insert size (180 bp to 16 kb) to generate scaffolds using both SOAPdenovo and SSPACE^32^. First, the gaps that resulted from the scaffolding were closed using GapCloser. Then, the PacBio long reads were mapped to the scaffold sequences using BLASR. PBJelly2 (ref. 39) was used to fill the gaps by generating consensus sequences of gap-spanning reads.

To remove potential bacterial contamination, the assembled contigs were first subjected to BLASTX searches against the NCBI non-redundant protein database. The contigs with the best-hit matches to bacteria were referred to as candidate bacterial contigs, and the contigs with the best-hit matches to *E. siliculosus* were referred to as authentic *Saccharina* contigs. For each contig, the GC content and the open reading frame (ORF) density (the total length of ORFs in the contig divided by the contig length) were calculated. The candidate bacterial contigs showing an ORF density of ≥60% or a biased GC content (>60 or <40%) were filtered. The assembly statistics are shown in Supplementary Table 2. This whole genome shotgun project can be accessed at http://124.16.129.28:8080/saccharina/.

### Transcriptome sequencing and analysis

RNA sequencing libraries were prepared with RNA samples from female gametophytes and sporophytes using the Illumina TruSeq RNA Sample prep Kit (Illumina). These RNA libraries were paired-end sequenced on an Illumina HiSeq 2000 sequencer. RNA sequencing reads were mapped against the *S. japonica* genome using TOPHAT v1.3.2 (ref. 40) with annotated exons to perform transcript-guided mapping. The GFOLD v1.0.5 (ref. 41) was employed to quantify gene expression levels with reads within or spanning exons. SOAPdenovo-Trans v 1.03 (ref. 42) was used to *de novo* assemble the RNA sequencing reads using default parameters. The *de novo* assembled transcripts were BLATed against the assembled genome to independently access genome completeness. The inGAP package^43^,^44^ was used to visualize and manually check read mapping of target regions.

### Detection and classification of repetitive elements

RepeatModeler (http://www.repeatmasker.org/RepeatModeler.html) and RepARK^45^ were employed to detect transposable elements (TEs) in the *S. japonica* genome using default parameters. RepeatModeler is a *de novo* repeat family identification and modelling package containing two *de novo* repeat-finding programs (RECON^46^ and RepeatScout^47^). RepARK (Repetitive motif detection by Assembly of Repetitive K-mers) is a wrapper script for constructing a repeat library from sequencing reads using Jellyfish and Velvet. After the raw repeat library was constructed with these two programs, CAP3 was run on the raw data using the parameters ‘-o 20, -i 30 -p 80 -s 400 -j 31' to assemble the TE fragments into full-length TEs and to remove redundancy, which was removed from the raw repeat library by cd-hit-est with the parameter ‘-c 0.8'. However, this newly built library still included plentiful short sequences (≤150 bp) produced by RepARK, which may have been meaningless fragments that would have significantly lengthened the CPU running time of RepeatMasker (http://www.repeatmasker.org). To address this problem, we only retained the sequences that were longer than 800 bp that were produced by RepARK in the new library and used the RepeatModeler-detected short repeats (≤800 bp) as substitute sequences. After applying RepeatMasker to mask the *S. japonica* genome with this new repeat library, we further simplified the library by removing the sequences with fewer than 50 hits in the *S. japonica* genome. The RepeatClassifier module in the RepeatModeler package was used to classify the identified repetitive elements based on Repbase.

### Insertion age of TEs in *S. japonica*

We first built a consensus sequence for each abundant TE using a combination of RepeatModeler and CAP3 (ref. 48). In total, four full-length RTE elements (RTE-1, RTE-2, RTE-3 and RTEX-1), six LTR elements (Gypsy-1, Gypsy-2, Copia-1, Copia-2, Copia-3 and ERV1), two Jockey elements (Jockey-1 and Jockey-2), one L1-1 and one CRE-1 element were reconstructed. For each TE, we aligned the matched hits identified by RepeatMasker to the consensus sequence and calculated the proportion of pairwise differences between these hits (*p*). The average number of substitutions per site for each fragmented repeat was estimated using the one-parameter Jukes–Cantor model (−3/4ln(1−4/3*p*)).

### Gene prediction and annotation

Homologue-based, *ab initio* and transcriptome-based approaches were integrated to predict protein-coding genes in the *S. japonica* genome. For the *ab initio* gene predictions, Augustus (version 2.5.5)^49^, GeneMarkES (version 3.0.1)^50^ and FGenesh^51^ were used to predict the protein-coding genes. The protein sequences of *Arabidopsis thaliana, Ectocarpus siliculosus, Chlamydomonas reinhardtii* and *Phaeodactylum tricornutum* were downloaded from Phytozome and used to align to the *S. japonica* genome with Exonerate v. 2.2.0 (ref. 52) using a Protein2Genome model to predict gene structures. The RNA sequencing data were mapped to the genome using Tophat^40^, and Cufflinks^53^ was employed to assemble transcripts to the gene models. The gene evidence predicted from the above three strategies was combined with EVidenceModeler^54^ into a non-redundant consensus of gene structures. We masked all of the TEs from the genome before gene prediction and filtered out all of the short-coding ORFs (<150 bp) supported by only *ab initio* methods.

The functional annotation of protein-coding genes was achieved using BLASTp (E-value 1E^−04^) against the NCBI non-redundant protein sequence database. The annotation information on the best BLAST hit derived from the database was transferred to the gene set. Motifs and protein domains were determined with InterProScan v5 (ref. 55) searches against the InterPro databases, including Pfam, PRINTS, PROSITE, PANTHER and SMART. The Gene Ontology IDs for each gene were obtained from the corresponding InterPro entries. All of the genes were mapped to KEGG proteins to determine the metabolic pathways.

### Identification of *S. japonica* gene families and phylogenetic analysis

We identified gene families in *S. japonica* by performing an all-against-all BLASTp search against the protein sequences of 25 algae and plants with available whole-genome information. Among these algae, 13 belonged to Chromalveolata (*S. japonica*, *E. siliculosus*, *Nannochloropsis gaditana*, *Nannochloropsis oceanica*, *Aureococcus anophagefferens*, *Phaeodactylum tricornutum*, *Thalassiosira pseudonana*, *Albugo laibachii*, *Phytophthora infestans*, *Pythium ultimum*, *Saprolegnia parasitica*, *Emiliania huxleyi* and *Guillardia theta CCMP2712*); 1 was Rhizaria (*Bigelowiella natans*); 1 was Glaucophyta (*Cyanophora paradoxa*); 5 were Rhodophyta (*Chondrus crispus*, *Cyanidioschyzon merolae*, *Galdieria sulphuraria*, *Porphyridium purpureum* and *Pyropia yezoensis*); 3 were Chlorophyta (*Chlamydomonas reinhardtii*, *Coccomyxa subellipsoidea* and *Volvox carteri*); and 2 were plants (*Arabidopsis thaliana* and *Physcomitrella patens*).

The global protein identities of each BLAST match were calculated using InParanoid^56^ to filter out matches exhibiting poor similarities (<20%) or poor gene coverage (<50%). Gene families were identified by MCL^57^ with the ‘inflation' option as 1.2. Gene numbers were counted for each family and for each species, and were compared by using the K-means algorithm in R. To obtain a robust species tree, redundant sequences (90% identity or more) from the same organism were removed using CD-HIT^58^, then, homologue clusters were predicted by comparing each pair of the 25 algal and plant genomes and further summarized by InParanoid and QuickParanoid. Subsequently, the clusters containing single-copy genes from each organism and clusters with species-specific duplications were selected for further consideration. For each cluster, multiple alignments were then performed using MUSCLE v3.8.31 (ref. 59) with the default parameters and were further trimmed using trimAl v1.4 (ref. 60) with the options ‘-gt 0.1 -resoverlap 0.75 -seqoverlap 80'. RAxML^61^ was employed to reconstruct a maximum-likelihood phylogenetic tree for each cluster with an evolutionary model specified as ‘PROTCATJTT' and to perform a bootstrap significance test with 100 replicates. TreSpEx^62^ was then applied to the tree of each cluster to evaluate taxa with long branches and to the trees of the 41 clusters with both long branch score heterogeneity and upper quartiles smaller than 30. The according clusters were selected for concatenation of their sequences within the same species into super genes.

A Dollo analysis was conducted on the homologue clusters of the 25 algal and plant proteomes using the Dollop tool in the PHYLIP package^63^ and custom Java scripts (available on request). To examine the evolutionary relationships between the duplicated homologues in the *S. japonica* genome before or after its divergence from *E. siliculosus*, synonymous (Ks) and non-synonymous (Ka) substitution rates were calculated using KaKs_Calculator^64^. A gene function-enrichment analysis for the clustered genes was performed using Fisher's exact test to compare all of the genes in the *S. japonica* genome.

### The vHPO and polysaccharide biosynthesis gene family analysis

Searches for vHPOs in the *S. japonica* genome were conducted with BLASTp (E-value<1e^−10^) using 8 vBPOs and 3 vIPOs present in *L. digitata* and *E. siliculosus* as reference sequences. All of the hits were determined by NCBI BLASTp using the default parameters. The phylogenetic tree was constructed by the neighbour-joining algorithm of the MEGA 5.0 program^65^, and a total of 1,000 bootstrap replicates were performed (Supplementary Table 3). Expression of vBPO and vIPO genes was investigated by real-time PCR in gametophytes, juvenile sporophytes and different tissues of adult sporophytes including holdfast, stipe, basal blades, middle blades and distal blades (Supplementary Table 4). The lowest expression of vBPO17 in the basal blade was set to 1. Average-linkage hierarchical clustering and heatmaps were generated in R Bioconductor using the heat map.2 function (omitting row and column dendrograms) in the gplots package of the R program (http://cran.r-project.org/web/packages/gplots/index.html). The analogous set of genes involved in the polysaccharide biosynthesis metabolism pathways for mannitol, trehalose, cellulose, laminarin, alginate, sulfate fucan and sucrose in the 14 algal genomes were identified and annotated based on KEGG and previous functional classifications^9^,^29^,^66^,^67^ (Supplementary Figs 12 and 15). To identify the CAZymes from *S. japonica* and distinguish the differences in cell wall polysaccharides from other algae, we performed CAZymes screening in *S. japonica* and the other 13 algal genomes (Supplementary Tables 6–8). All of the putative proteins were searched against entries in the CAZy database using the dbCAN Web server^68^, in which HMMer^69^ was used to query against a collection of custom-made hidden Markov model profiles that were constructed for each CAZy family.

### Identification of SNPs and InDels from resequenced *Saccharina* genomes

The paired-end reads from the 14 resequenced samples (C2, C3, C5, C6, C8, C11, C12, C13, C15, C17, B, F, G and W) were aligned to the draft assembly using BWA v.0.7.5. We employed the default parameters to align the reads, with the exception of the ‘-q 15' parameter, which was used to trim the low-quality regions at the 3′ ends of the reads before mapping. Bcftools in the SAMtools^50^,^70^ package was employed to call all of the raw variant locations using the parameters ‘bcftools view –vcg'. The locations of raw variants in C8, C11, C13, C15 and C17 were filtered using the parameters ‘vcfutils.pl varFilter -d 10 -D 100 -Q 20', whereas the remaining nine samples were filtered using the parameters ‘vcfutils.pl varFilter -d 3 -D 40 -Q 20'. Once a list of putative variant locations had been constructed, we used the mpileup command in SAMtools to call the genotypes for each sequenced individual.

The paired-end reads from the two distantly related species, E and C14 were *de novo* assembled separately using SOAPdenovo^38^. The assembled contigs were scaffolded using the paired-end reads. GAPcloser was employed to close the gaps in the scaffolds. NUCmer was used to align the assembled sequences to the *S. japonica* Ja assembly. After filtering the multiple mapped regions, NUCmer was employed to call single-nucleotide variants and short InDels.

### Phylogenetics and population structure of *Saccharina*

Homozygous single-nucleotide polymorphism (SNPs) were used to calculate the genetic distances between different *S. japonica* accessions. The neighbour-joining method in MEGA5 was applied to construct a phylogenetic tree based on the p-distance method with 1,000 bootstrap replicates.

We used STRUCTURE software (version 2.3.2) to investigate population structure across different values of *K*, representing the number of putative ancestral clusters of allelic similarity. We employed an admixture model with correlated allele frequencies to assign individuals to the *K* clusters. A 10,000 step burn-in period for Monte Carlo Chain searches, followed by 20,000 replicate runs, was applied at each *K* from 2 to 6.

## Author contributions

N.Y. and F.Z. conceived the project, designed content and managed the project; X.Z. and D.L. coordinated the project; X.Z. and X.F. prepared DNA and RNA samples; D.X., D.W., Y.W., Z.G. and Z.G. provided algal materials and conducted experiments; J.W. constructed DNA libraries and performed sequencing; F.Z. and P.J. performed raw data analysis; F.Z. and W.S. performed the genome assembly; Y.Z. identified and annotated repeats; J.Q. and F.Z. performed transcriptome analysis, gene prediction and annotation; X.F. performed mitochondrial and chloroplast genome assembly and annotation; X.Z., X.F., G.Y., L. Z., J.Q. and F.Z. performed comparative genomic analyses; F.Z. performed read mapping and SNP identification; and M.M. and F.Z. performed phylogenetic and population genetic analyses. X.Z., F.Z., J.Q., X.F., M.M., Y.Z. and Y.N. wrote the manuscript. Z.Z. and S.C. revised the manuscript. All authors commented on the manuscript.

## Additional information

**Accession codes.** This whole genome shotgun project has been deposited in GenBank under the accession code JXRI00000000.

**How to cite this article:** Ye, N. *et al*. *Saccharina* genomes provide novel insight into kelp biology. *Nat. Commun*. 6:6986 doi: 10.1038/ncomms7986 (2015).

## Supplementary Material

Supplementary Information Supplementary Figures 1-15, Supplementary Tables 1-8, Supplementary Notes 1-2, Supplementary Methods and Supplementary References

Supplementary Data 1 The 41 ortholog genes used for constructing phylogenetic tree

Supplementary Data 2 Gene families predicted from the S. japonica and the other 24 genomes

Supplementary Data 3 Gain and loss gene families obtained using Dollo analysis

Supplementary Data 4 Selected genes with decreased Tajimas D values in cultivated S. japonica individuals

Supplementary Data 5 Selected genes with elevated Tajimas D values in cultivated S. japonica individuals

Supplementary Data 6 Selected genes with decreased Tajimas D values in wild S. japonica individuals

Supplementary Data 7 Selected genes with elevated Tajimas D values in wild S. japonica individuals
