# Supplementary material for: Saccharina genomes provide novel insight into kelp biology
Source: Nat Commun. 2015 Apr 24;6:6986. doi: 10.1038/ncomms7986 (PMC4421812; doi:10.1038/ncomms7986)
Supplement: Supplementary Information — Supplementary Figures 1-15, Supplementary Tables 1-8, Supplementary Notes 1-2, Supplementary Methods and Supplementary References [file ncomms7986-s1.pdf]

Supplementary Figures

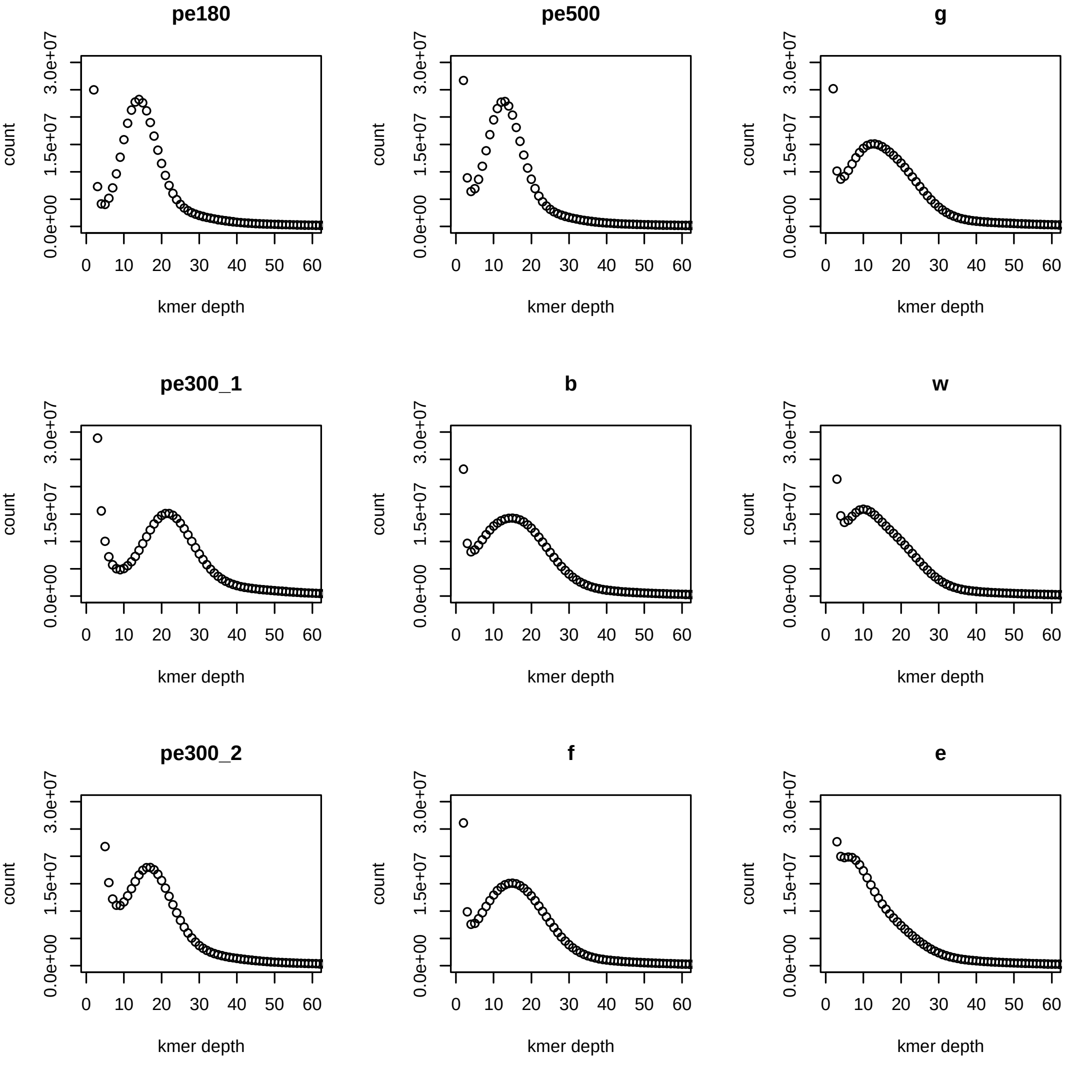

Supplementary Figure 1. Kmer depth analyses. "pe180", "pe300_1", "pe300_2", and "pe500" represents the kmer frequency distribution of four short insert libraries in *S. japonica* JA, respectively. "b", "f", "g", "e", and "w" represents the kmer frequency distribution of the sequencing data in four wild individuals and one cultivated *S. japonica* individual. The left peak at low frequency represents kmers containing random sequencing errors, whereas the right long tail with high kmer depth likely results from repetitive sequences. The middle distribution represents error-free data and the peak is considered as kmer coverage. The genome size was calculated based on the coverage estimate and total number of non-error kmers.

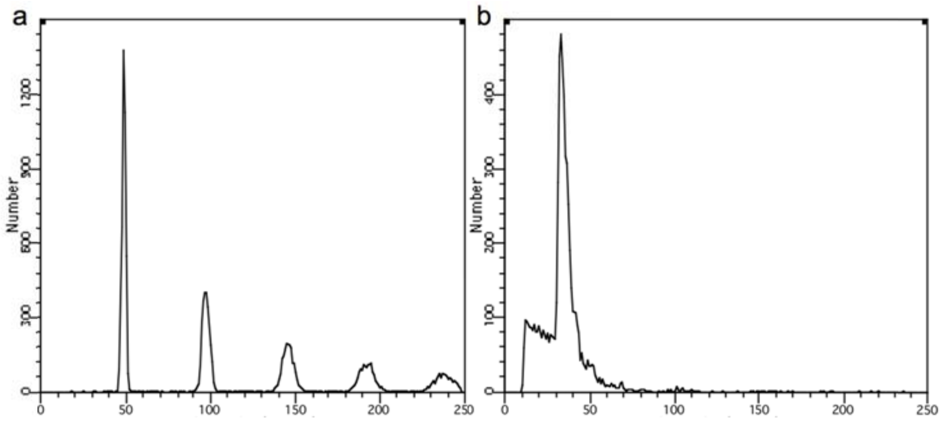

Supplementary Figure 2. Genome size determination of *S. japonica* by flow cytometry (FCM) analysis of fluorescently stained nuclei using chicken erythrocytes as an internal standard. a, Chicken erythrocytes. b, single cells of *S. japonica* gametophytes. X and Y axis show the relative fluorescence and the number of nuclei, respectively.

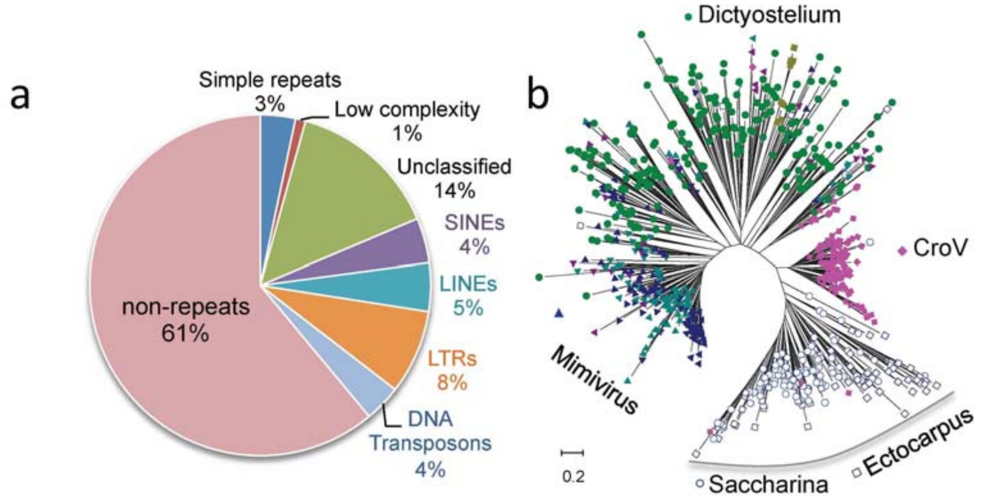

Supplementary Figure 3. Repetitive elements in *S. japonica*. (A) The fraction of various types of repetitive elements in the *S. japonica* JA genome. (B) A phylogenetic tree of FNIP repeats in *S. japonica* and all of the currently available genomes.

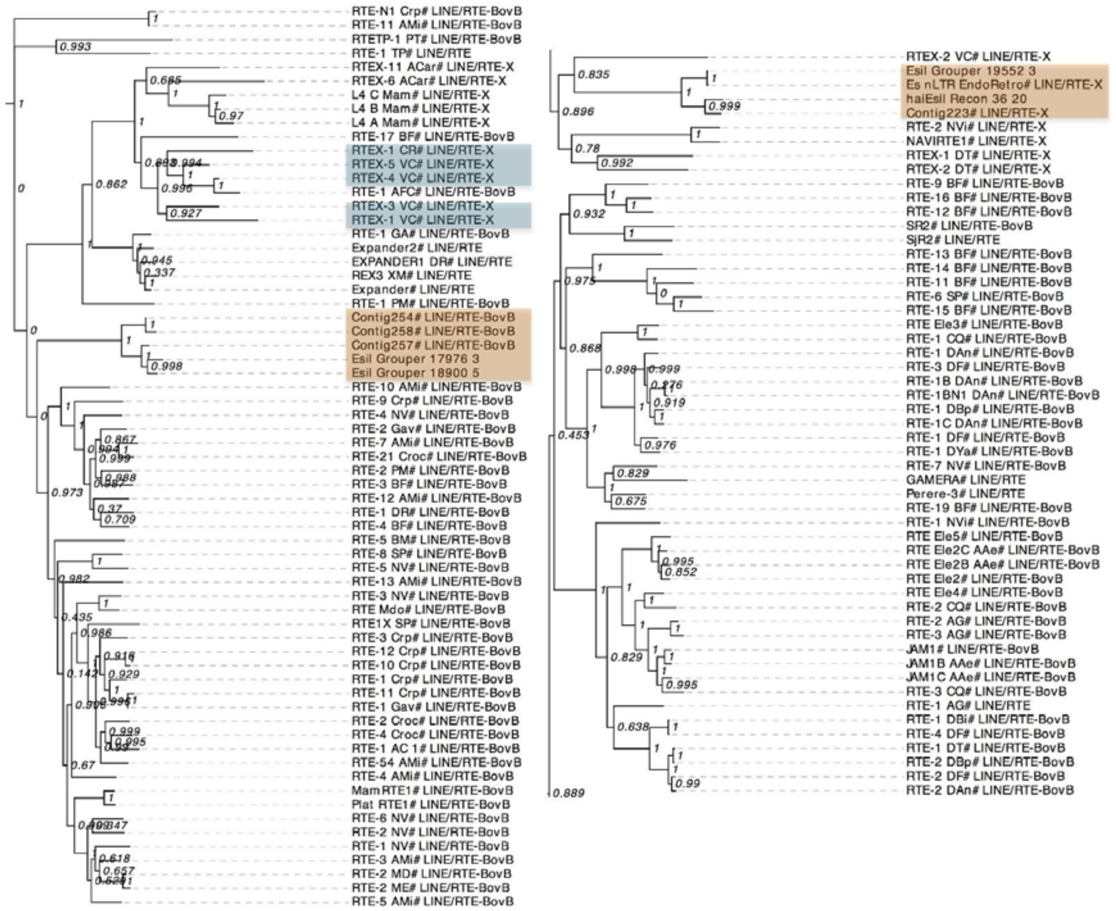

Supplementary Figure 4. Phylogenetic analysis of RT-domains in brown algal RTEs and known RTEs in Repbase. RTEs from brown algae and green algae are highlighted in brown and green, respectively.

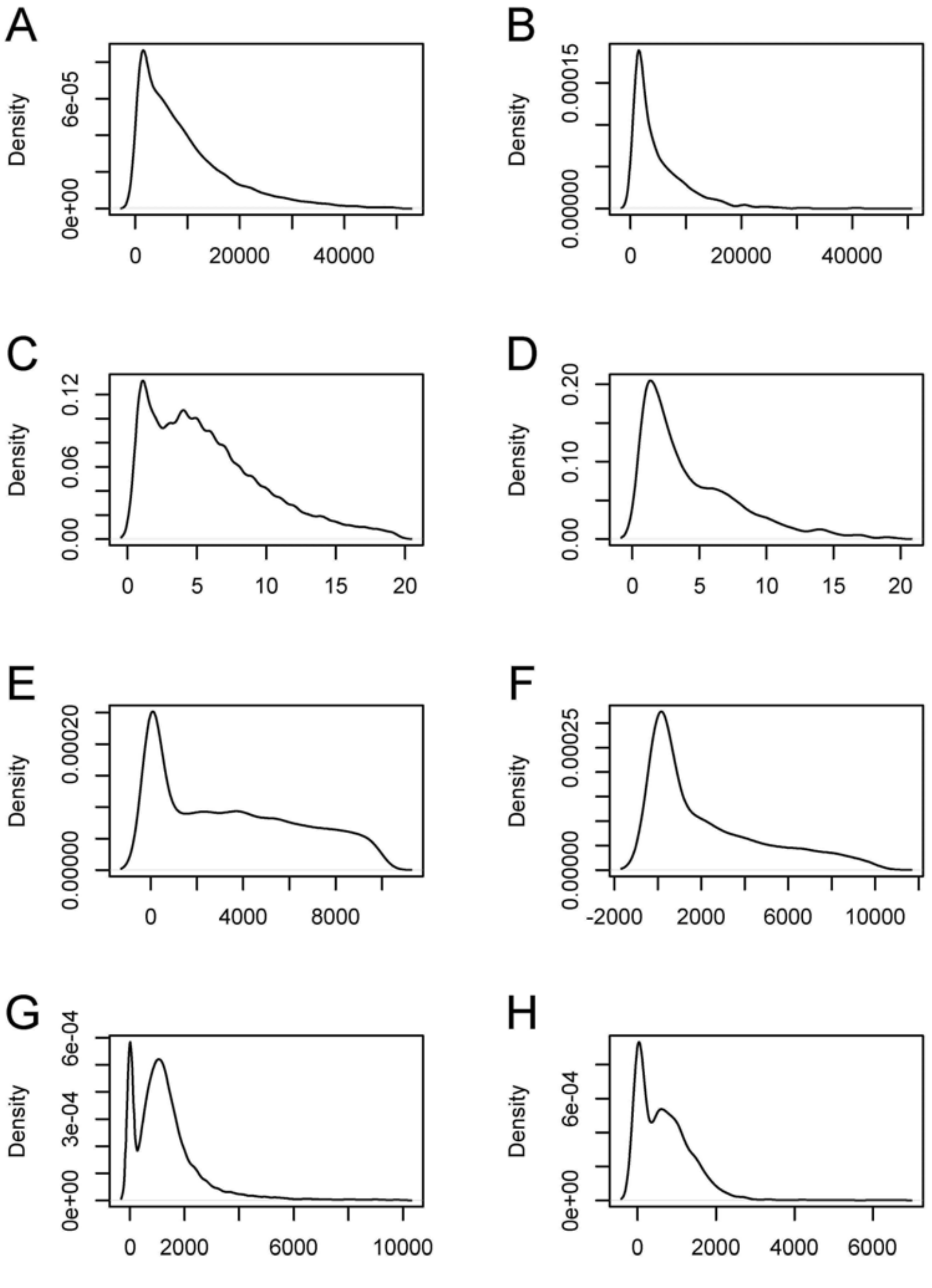

Supplemental Figure 5. Comparison of genes with RNA-seq data support (left figures) or without support (right figures) in aspect of (A and B) gene length, (C and D) exon number, (E and F) sum of intron length, and (G and H) average of intron length. A total of 1,534 of 1,738 genes without RNA-seq support have homologues in the *Ectocarpus* genome.

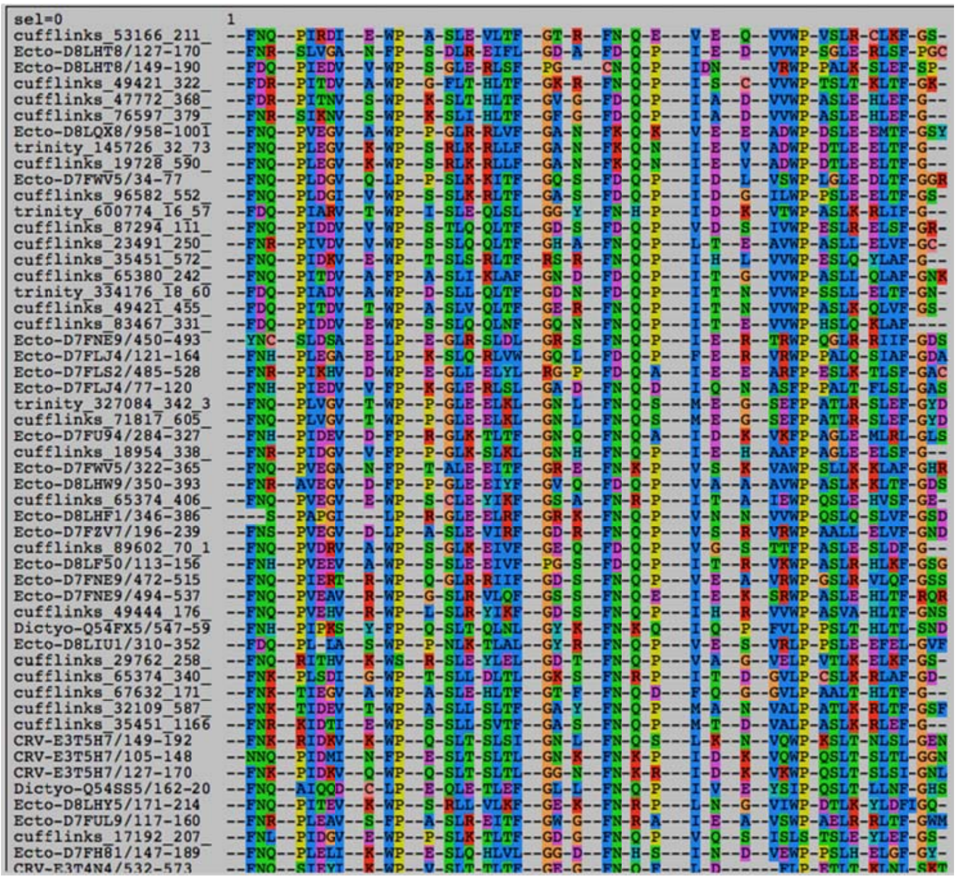

Supplementary Figure 6. The alignment of FNIP domains. To reduce the figure size, only a subset of sequences was shown.

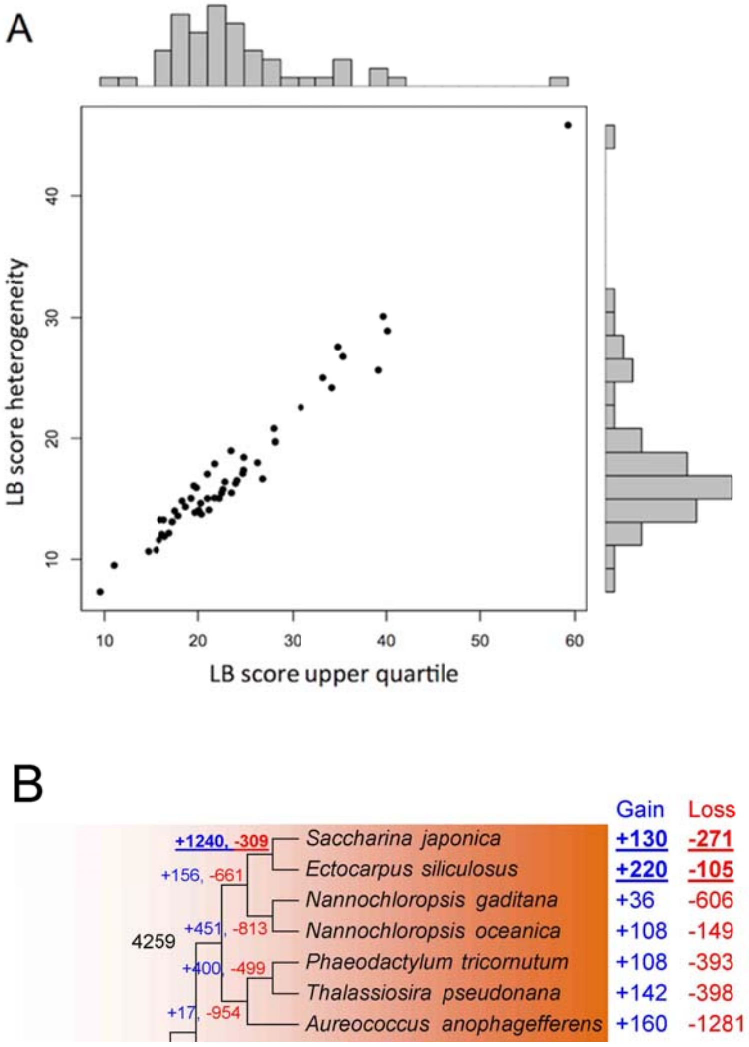

Supplemental Figure 7. Evolutionary analysis of 25 organisms from Chromalveolata, Rhizaria, Glaucophyta, Rhodophyta, Chlorophyta and higher plants. (A) Illustration of branch length heterogeneities and long branch scores for maximum-likelihood trees of single orthologs containing a single copy gene for each organism and those with species-specific gene duplications. (B) The gene family gains and losses in the genomes of seven heterokontic algae. The number of gene family gains and losses for each branch are labeled in blue and red, respectively. The underlined and bold numbers were used for PFAM enrichment analysis in Fig. 2B.

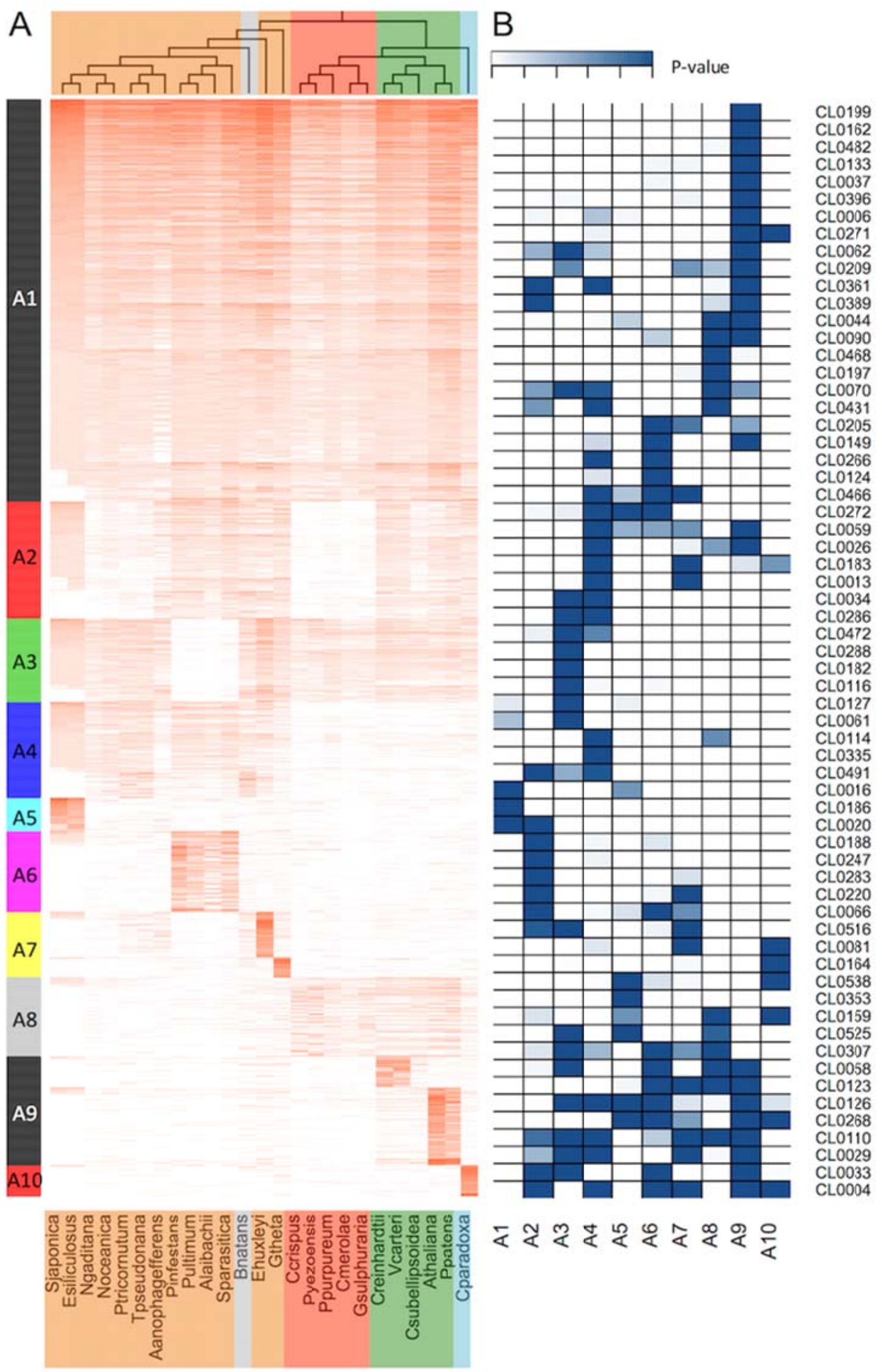

Supplemental Figure 8. Comparison of gene abundance among 25 organisms for 4780 families containing 10 or more genes. (A) Families were clustered into 10 groups based on the gene abundance in each species by using K-means method; each row represents a family, and each column represents one species. (B) The domain-based annotation of clustered families in (A). Fisher's exact test of the domain enrichment of the families was compared against the total number of annotated domain in both of the genomes.

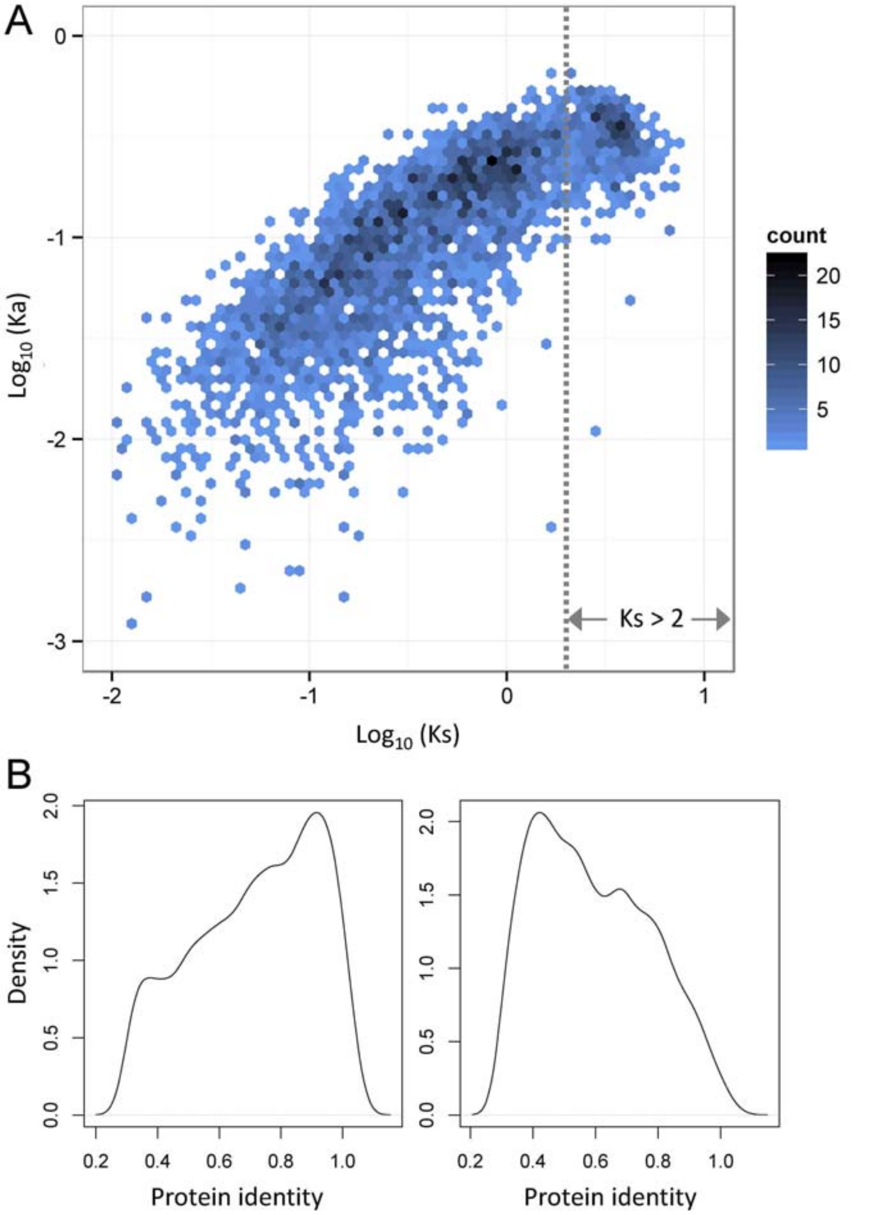

Supplemental Figure 9. (A) Analysis of non-synonymous (Ka)/synonymous (Ks) for *S. japonica* genes against their most similar *S. japonica* homologs within the same family with gene expansion in *S. japonica*. The logarithmic values of Ka and Ks with base 10 were displayed. Ks values larger than 2 (represented by a vertical doted line) were considered as saturated substitution rates. (B) Statistics of Protein identities of *S. japonica* gene pairs (left panel) and those between *S. japonica* and *E. siliculosus* (right panel).

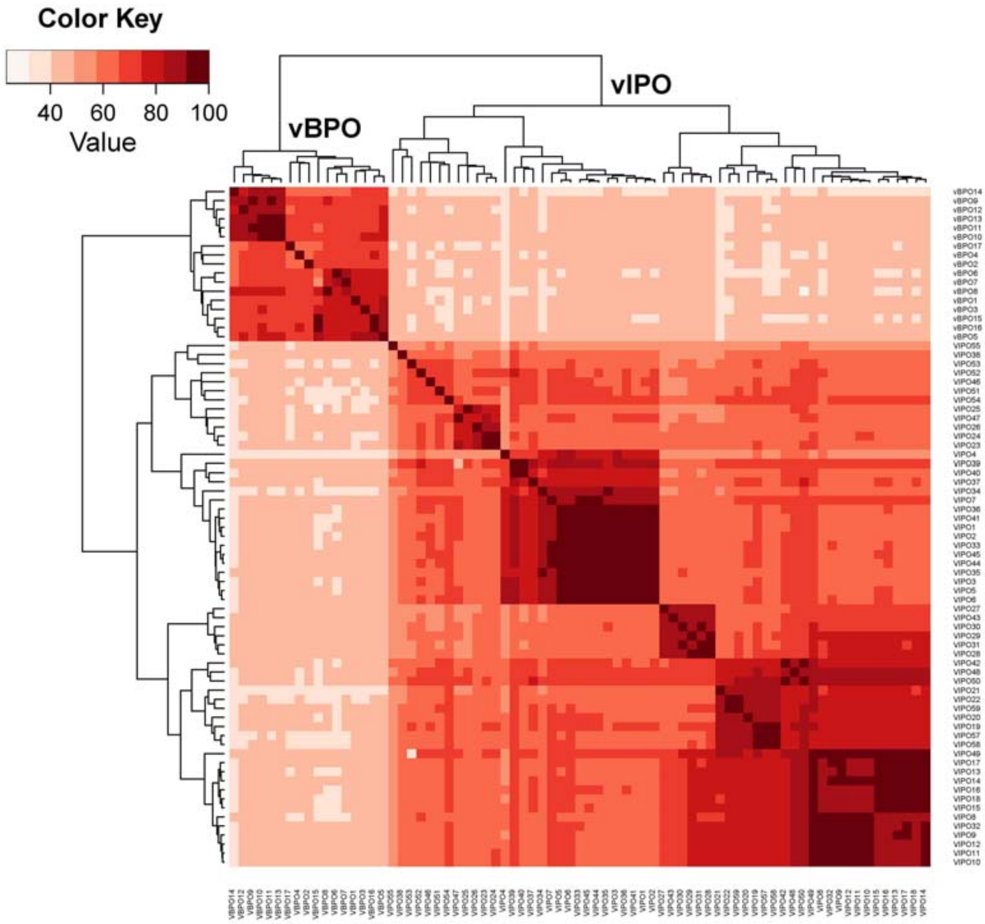

Supplementary Figure 10. Diversification of vBPO and vIPO genes from *S. japonica*. A heat-map representation of the hierarchical clustering analysis of 15 vBPO and 42 vIPO genes obtained from *S. japonica*. The analysis is performed using sequence similarity scores from pairwise alignments. The dendrogram illustrates the sequence identites between vBPOs and vIPOs.

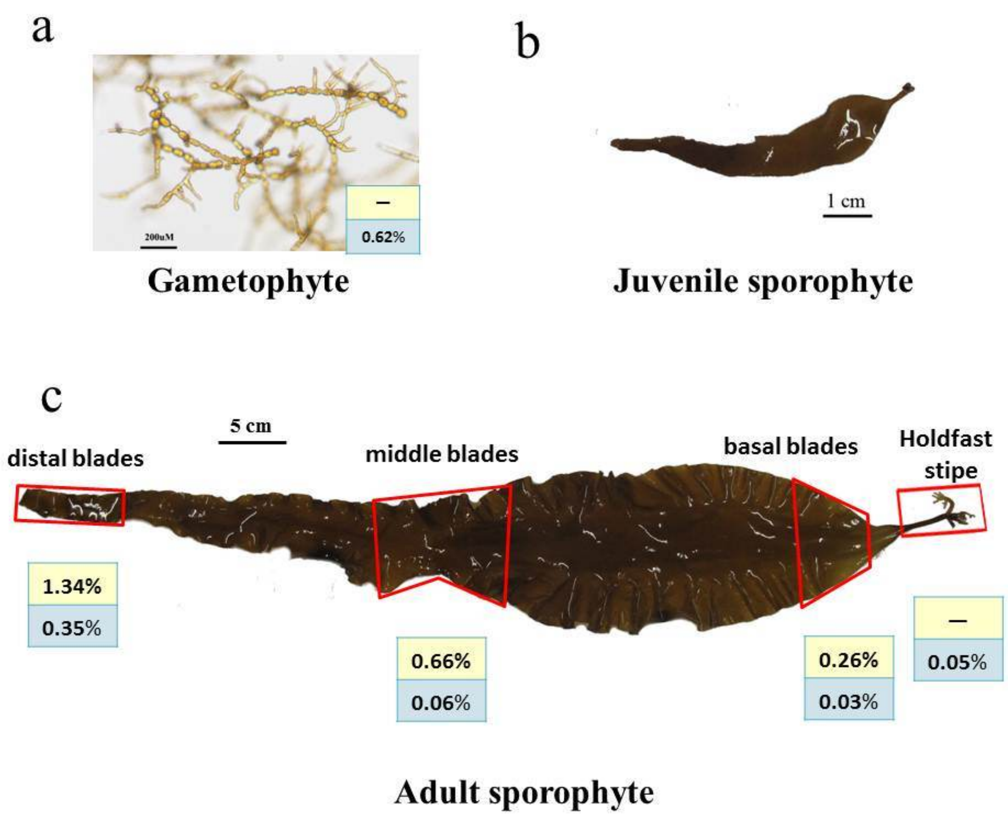

Supplementary Figure 11. Different life stages of *S. japonica* and Iodine contents in adult sporophyte. a) Gametophyte and Iodine contents; and b) Juvenile sporophyte used for vHPO gene expression investigation; c) Iodine contents in basal blades, middle blades and distal blades were determined by iodometry and were shown in the light yellow boxes; Iodine contents in gametophyte, three blade parts, holdfast and stipe were determined by Scanning Electron Microscopy (SEM) analyses and were shown in the light blue boxes.

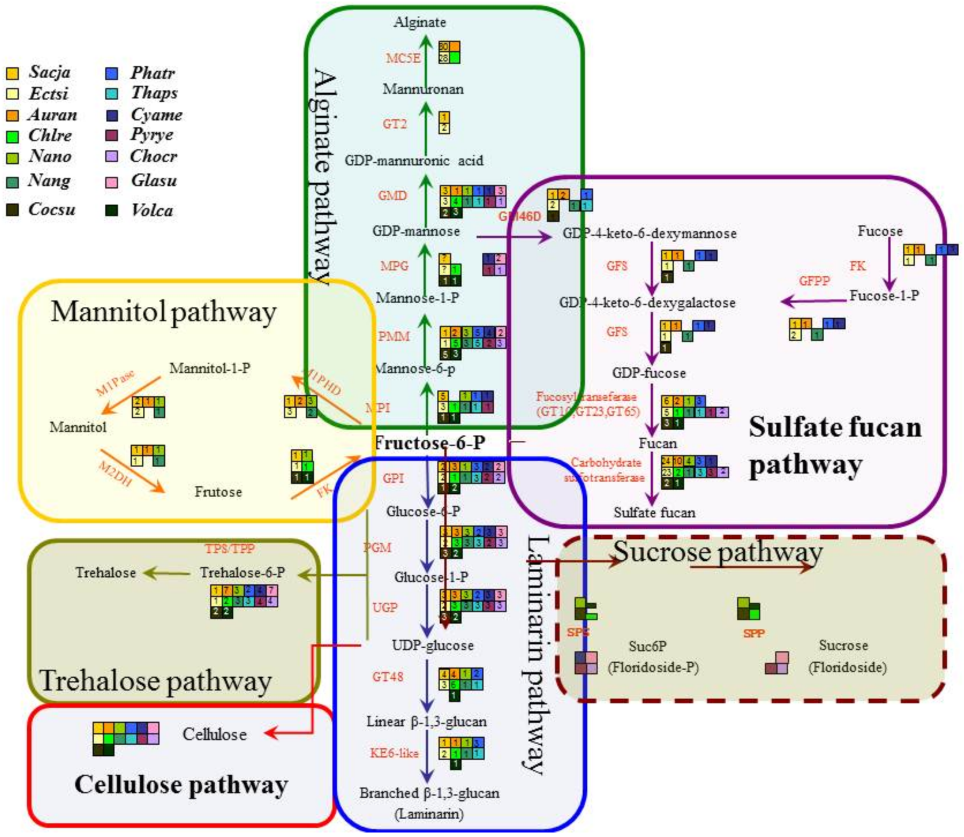

Supplementary Figure 12. Reconstruction the carbon metabolism pathways in 14 algae genomes. M1PDH, mannitol-1-phosphate 5-dehydrogenase; M1Pase, mannitol-1-phosphatase; M2DH, mannitol-2-dehydrogenase; FK, fructokinase; GPI, glucose-6-phosphate isomerase; PGM, phosphoglucomutase; UGP, UDP-glucose-pyrophosphorylase; GT48, b-1,3-glucan synthases (family GT48); KRE6-like proteins, putative 1,6-b-transglucosylases (family GH16); UDP, uridine diphosphate; GDP, guanosine diphosphate; TPS, trehalose-phosphate synthase; TPP, trehalose-phosphate phosphatase; GMD, GDP-mannose 6-dehydrogenase; MC5E, mannuronate C5-epimerase; MPG, mannose-1-phosphate guanylyltransferase; MPI, mannose-6-phosphate isomerase; MS, mannuronan synthase; PMM, phosphomannomutase; FK, l-fucokinase; GFPP, GDP-fucose pyrophosphorylase; GFS, GDP-l-fucose synthetase; GM46D, GDP-mannose 4,6-dehydratase. *Nanoc=Nannochloropsis oceanica*，*Nanga= Nannochloropsis gaditana*, *Auran= Aureococcus anophagefferens*, Ectsi= *Ectocarpus siliculosus*, *Sacja= Saccharina japonica*, *Thaps= Thalassiosira pseudonana*, *Phatr= Phaeodactylum tricornutum*, *Chocr=Chondrus crispus*, *Cyame= Cyanidioschyzon merolae* , *Pyrye=Pyropia yezoensis*, *Galsu=Galdieria sulphuraria*, *Chlre= Chlamydomonas reinhardtii*, *Cocsu=Coccomyxa subellipsoidea*, *Volca=Volvox carteri.*

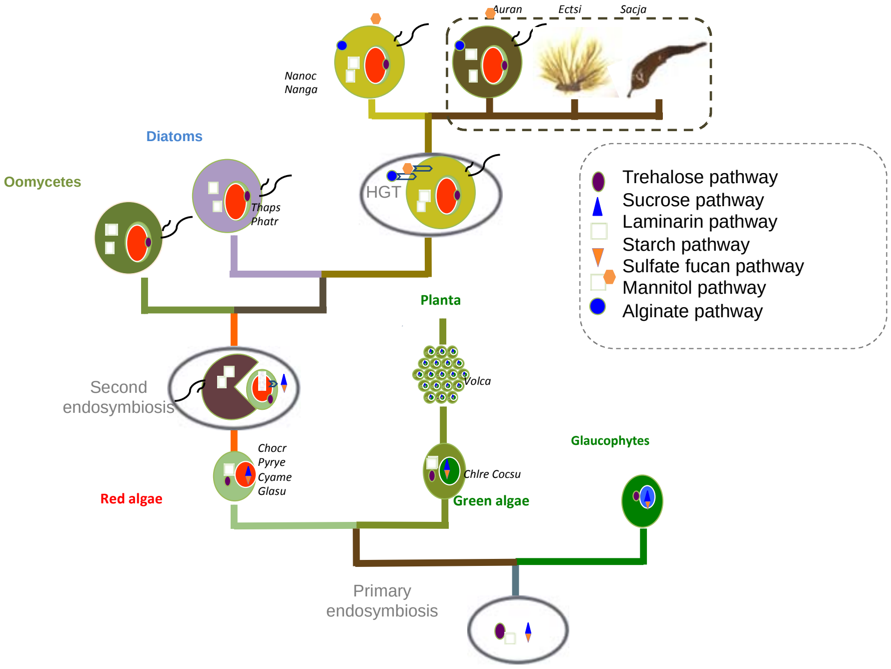

Supplementary Figure 13. Proposed scenario for the evolution of polysaccharide biosynthesis and metabolism in green algae, red algae and stramenopiles. The sucrose and starch pathway in red algae, green algae and Glaucophytes evolved from ancient cyanobacteria via primary endosymbiosis. Brown algae, and heterokonts in general, acquired ancient red algae through secondary endosymbiosis, during which the sucrose and starch pathway was lost, and the laminaran pathway was introduced. The genes involved in the mannitol and alginate pathway in brown algae are very similar to those found in Actinobacteria, indicating the occurrence of horizontal gene transfer (HGT) events. The HGT event showed key genes related to the mannitol and alginate pathway were acquired by the common ancestor of brown algae, Eustigmatophyceae (*Nannochloropsis*) and Pelagophyceae through multiple HGT events. Other HGT events related to polysaccharide biosynthesis were not considered here.
Nanoc=Nannochloropsis oceanica， *Nanga= Nannochloropsis gaditana*, *Auran= Aureococcus anophagefferens*, Ectsi= *Ectocarpus siliculosus*, *Sacja= Saccharina japonica*, *Thaps= Thalassiosira pseudonana*, *Phatr= Phaeodactylum tricornutum*, *Chocr=Chondrus crispus*, *Cyame= Cyanidioschyzon merolae* , *Pyrye=Pyropia yezoensis*, *Galsu=Galdieria sulphuraria*, *Chlre= Chlamydomonas reinhardtii*, *Cocsu=Coccomyxa subellipsoidea*, *Volca=Volvox carteri.*

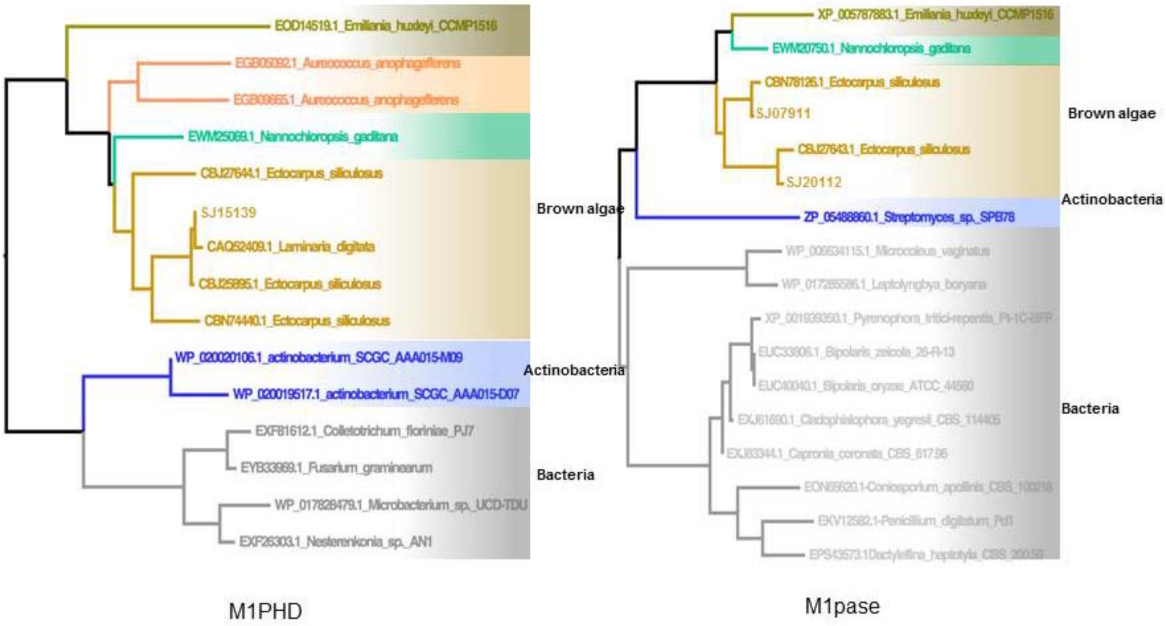

Supplementary Figure 14. Phylogenetic analysis of enzymes M1PHD and M1pase corresponding in mannitol synthesis. The abbreviated words begin with and “SJ” in the tree stand for the protein ids for *S. japonica*. Actinobacteria and Bacteria taxon names were represented by the NCBI protein accession numbers.

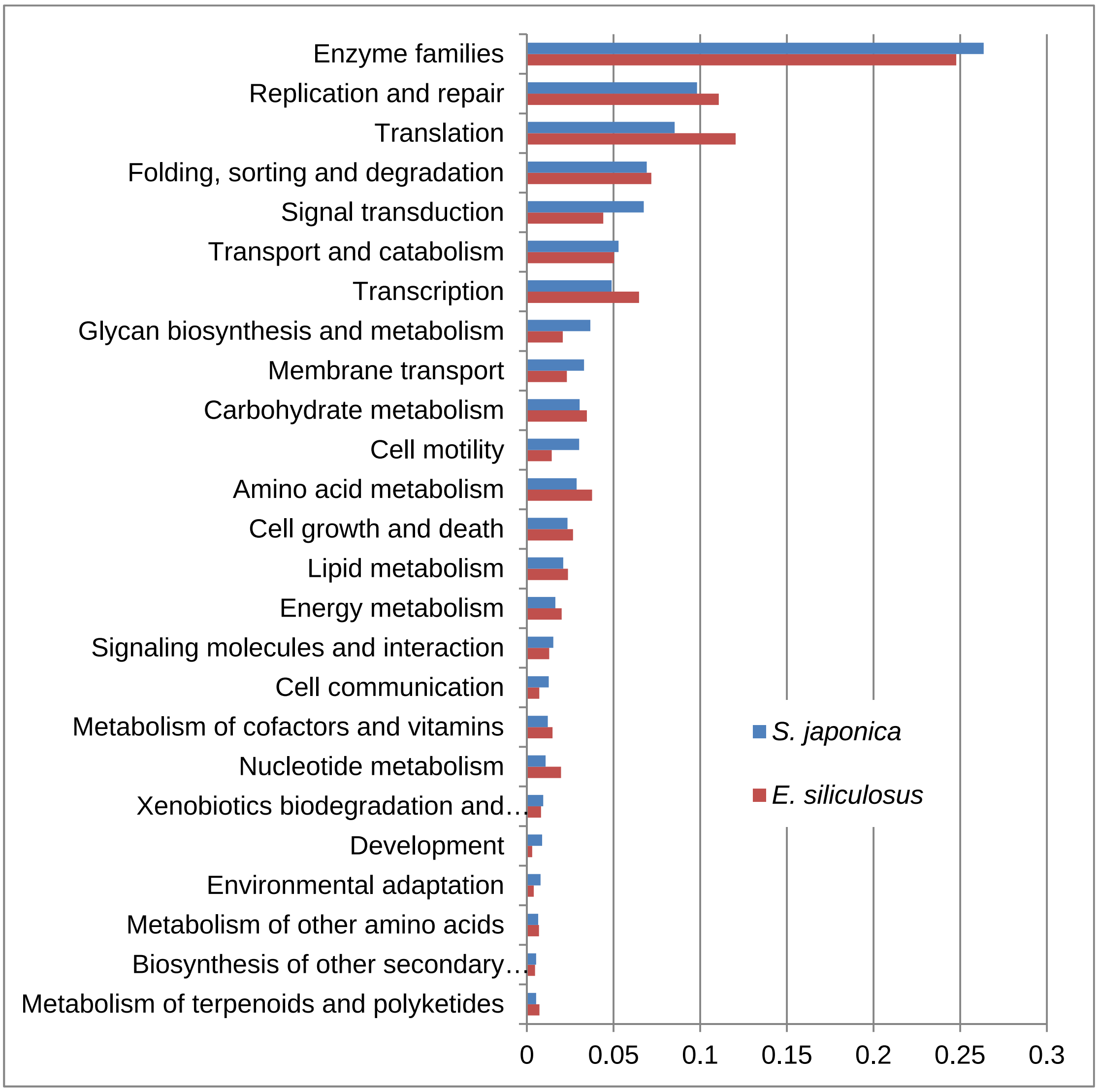

Supplementary Figure 15. The KEGG distribution of proteins in *S. japonica* and *E. siliculosus* respectively. The x-axis indicates the percentage of a specific category of genes in the species. The signal transduction, glycan biosynthesis and metabolism, membrane transport, cell communication and development pathways were enriched in *S. japonica* when compared to *E. siliculosus*.

Supplementary Tables

Supplementary Table 1. Sample and sequencing statistics

ID	Taxonomy	Type	Location	Sequencer	Library Size	Read length	#Reads	DOC[3]	pDOC[4]
**Ja**	*S. japonica*	FG[1]	Qingdao, China	Illumina	180bp	101bp	173,745,638	32X	29X
				Illumina	300bp	101bp	343,890,974	64X	96X
				Illumina	500bp	101bp	229,303,234	42X	106X
				Illumina	800bp	101bp	12,440,146	2X	9X
				Illumina	3kb	101bp	55,051,984	10X	153X
				Illumina	5kb	100bp	26,018,116	5X	120X
				454	8kb	373bp	1,367,320	0.9X	21X
				454	16kb	349bp	1,194,418	0.8X	36X
				PacBio	/	5kb	1,928,517	20X	/
**C2**	*S. japonica*	FG	Dalian, China	Illumina	300bp	101bp	246,683,234	45X	68X
**C3**	*S. japonica*	FG	Lianjiang, China	Illumina	300bp	101bp	276,867,214	51X	77X
**C5***	*S. longissim*	FG	Japan	Illumina	300bp	101bp	212,293,176	39X	59X
**C6***	*S. japonica*	FG	Japan	Illumina	300bp	101bp	281,665,262	52X	78X
**C8**	*S. japonica*	FG	Rongcheng,China	Illumina	300bp	101bp	94,908,970	17X	26X
**C11**	*S. japonica*	FG	Dalian, China	Illumina	300bp	101bp	98,206,724	18X	27X
**C12**	*S. japonica*	FG	Qingdao, China	Illumina	300bp	101bp	230,401,208	42X	64X
**C13**	*S. japonica*	FG	Rongcheng,China	Illumina	300bp	101bp	82,852,948	15X	23X
**C14***	*S. latissima*	FG	Germany	Illumina	300bp	101bp	254,911,106	47X	71X
**C15***	*Saccharina* sp.	FG	Russia	Illumina	300bp	101bp	71,700,190	13X	20X
**C17***	*Saccharina* sp.	FG	Russia	Illumina	300bp	101bp	82,773,558	15X	23X
**B***	*Saccharina* sp.	SP[2]	Russia	Illumina	300bp	100bp	158,892,558	29X	44X
**E***	*Saccharina* sp.	SP	Russia	Illumina	300bp	100bp	138,363,232	25X	38X
**F***	*Saccharina* sp.	SP	Russia	Illumina	300bp	100bp	166,419,490	31X	46X
**G***	*Saccharina* sp.	SP	Russia	Illumina	300bp	100bp	206,223,678	38X	57X
**W***	*S. japonica*	SP	Rongcheng,China	Illumina	300bp	100bp	157,566,200	29X	44X

Note: FG[1] indicates Female Gametophyte; SP[2] indicates Sporophyte; DOC[3] indicates depth of coverage; pDOC[4] indicates physical depth of coverage; * indicates wild species.

Supplementary Table 2. *S. japonica* genome assembly statistics

Statistics	*S. japonica*
Number of scaffolds	13,327
Total size of scaffolds	543,425, 768
Longest scaffold	1,469,055
Number of scaffolds > 500 nt	13,327
Number of scaffolds > 1K nt	8,872
Number of scaffolds > 10K nt	4,287
Number of scaffolds > 100K nt	1,575
Mean scaffold size	40,776
Median scaffold size	2,394
N50 scaffold length	252,007
L50 scaffold count	628
scaffold %A	24.90
scaffold %C	24.58
scaffold %G	24.56
scaffold %T	24.88
scaffold %N	1.09
Percentage of assembly in scaffolded contigs	85.5%
Percentage of assembly in unscaffolded contigs	14.5%
Average number of contigs per scaffold	2.2
Average length of break (>25 Ns) between contigs in scaffold	444
Number of contigs	29,670
Number of contigs in scaffolds	19,873
Number of contigs not in scaffolds	9,797
Total size of contigs	537,640,579
Longest contig	648,295
Number of contigs > 500 nt	28,287
Number of contigs > 1K nt	22,356
Number of contigs > 10K nt	10,891

Number of contigs > 100K nt	992
Mean contig size	18,121
Median contig size	4,429
N50 contig length	58,867
L50 contig count	2,495

Supplementary Table 3. Sequences used for vHPO phylogenetic analysis

Abbreviation	Accession Number
Laminaria digitata VBPO1	CAD37191.1
Laminaria digitata VBPO2	CAD37192.1
Laminaria digitata VBPO3	CAQ51441.1
Laminaria digitata VBPO4	CAQ51442.1
Laminaria digitata VBPO5	CAQ51443.1
Laminaria digitata VBPO6	CAQ51444.1
Laminaria digitata VBPO7	CAQ51445.1
Laminaria digitata VIPO1	CAF04025.1
Laminaria digitata VIPO3	CAQ51446.1
Ectocarpus siliculosus VBPO	CBN73942.1
Corallina pilulifera VBPO1	BAA31261.1
Corallina Pilulifera VBPO3	37928252
Corallina officinalis VBPO1	AAM46061.1
Corallina officinalis VBPO2	9256866
Corallina pilulifera VBPO2	BAA31262.1
Chondrus crispus VBPO	XP_005719435.1
Gracilaria changii VBPO1	AGE00855.1
Laurencia nipponica VBPO	BAP16518.1
Pyropia yezoensis VBPO	BAQ02347.1
Mesoflavibacter zeaxanthinifaciens VBPO	WP_010516409.1
Citreicella sp. 357 VBPO1	WP_009505876.1
Synechococcus sp. WH 8020 VBPO1	ADO20318.1
Synechococcus sp. CC9311 VBPO2	YP_731869.1
Alteromonas sp. SN2 VBPO1	YP_004466264.1
Acaryochloris sp. CCMEE 5410 VBPO	WP_010471680.1
Acaryochloris marina VBPO2	WP_012161787.1
Acaryochloris marina VBPO	WP_012165216.1
Acaryochloris marina MBIC11017 VBPO	YP_001519261.1
Leptolyngbya sp. VBPO3	WP_023074445.1

Magnaporthe oryzae VCPO2	EHA56362.1
Gaeumannomyces graminis VCPO	EJT71764.1
Magnaporthe oryzae VCPO1	ELQ63332.1
Pyrenophora triticirepentis VCPO	XP_001933850.1
Alternaria didymospora VCPO	CAA72622.1
Curvularia inaequalis VCPO	CAA59686.1
Zobellia galactanivorans VIPO1	YP_004735706.1
Zobellia galactanivorans VIPO2	YP_004736527.1

Supplementary Table 4. Primers for real-time PCR of vHPO in *S. japonica*

Target	Forward	Reverse
beta tubulin		
Tublin	AGGTGGACGAGCAGATGC	GAGGCCCTTAGGAGGTACG
vanadium-dependent iodoperoxidase		
vIPO2	GCAAACGACCTTCCAAAC	CGTTTCACCGTGTCCTCC
vIPO5	CATCTCACCCGTCGTACC	GAAGCCCTTCTCCAGTCC
vIPO6	CGGCAAACGACCTTCG	TATCCTCCCTTTCCATCTCC
vIPO8	ACCAACTCATCCGTCGTAC	AACACGGGGTCATTGAAGC
vIPO9	CGCCTTAACCATCCCTAGC	ACTGGGAGAAAGGTACGTC
vIPO10	GGCCAGACGATCAGACTCG	GGTCAGCTCCCATGAACG
vIPO14	CTTTTCACCGGAGCAAGC	CGAATTACTGCTCTCCAAGG
vIPO16	CCTTGACGAGGGACGTACC	TGTTCATATCGGCGAAACC
vIPO17	TTCTCATCGAGGAAGGAGC	CCTCATAACATGCGACACG
vIPO18	ATTTTCACCGGAGCAAGC	AAAGACTGCCCTCCAAGG
vIPO19	AAGGATTTCACTCTGGACTC	GTATGAAGAGCCATTCGTC
vIPO21	GGCACGACTGATCAAACG	CTGGGAGAAAGGTACGTCC
vIPO22	GGAAGACCCGGTGAAGC	AGAATCAGGGCAGCACG
vIPO23	AGGAAGTCTCGGAGCTGC	CATTCATGGTCGTCCAGG
vIPO24	ATTCAACGGCCCCTACC	CCCAGAATCCTCATGACG
vIPO25	CATCCACAACGTCCTACTC	CTACCCTCTCCAGCAATTCC
vIPO26	TTTCAACGGCCCCTACC	GCGCTCCCCAGAATCC
vIPO28	GGAACCATCCACAACATCC	CCACCTTGCTGAACAATAGG
vIPO29	ATGCCATAACCATCCCTGC	GGCCATCCAGTACACCTCC
vIPO30	CTGATCCTGATCGAGCAAG	GGAGATGCCGTAGTTGACG
vIPO31	CGACTCACCCATCGTACC	TCCAGCCCAACAAGAGC
vIPO32	GGAGCTATCGGGGAATCG	CAGAGCACTGCCGTCTAAGG
vIPO33	AAGGGATGTGCCTTTCAAC	CCCATTCATGTTAGCGAAAC
vIPO34	AAGGCGACTTTGACAAGTG	CAACGGCGAGACCTCC
vIPO35	CGACAAGAAATGCAGCTCG	CAGAACCCTGTACTGCCAGC
vIPO36	ATCCCACCCGTCGTACC	TCGTTGAAGCATTCGAACC
vIPO37	TCCTATACGGCAACCTCG	CCTAGACCTCCTGCAAGTCC
vIPO38	GCCAAGCAGACTACCTACA	TTCTCGTCGTCGTCCTCC
vIPO39	GCAGACGACCTTCAAAACC	GCTTCTCCGAGTCCTCAGC
vIPO40	CCACAGTCCCAACTGTTCC	GTCACCGTCAACGAGTCC
vIPO41	GGTAAACAGCTTCGCAAAC	GTTGAAAGGCACATCTCTCG
vIPO42	CCTTGACGAGGGATGTACC	GTTCATGTTGGCGAAACC
vIPO44	TTCGTAAACGACAAGAAAT	CCCTAAACTGACAGCTTCAG
VIPO46	CAACAAACTAGCCACCAAC	CTCCCCGAGATGCAACC
vIPO47	AGGGAGAACTCAACAAAAT	CTTCCTCGCTGTCGAACC

vIPO48	CTTCCCCACGACCACC	TCGCACTTGTCAAAGTCTC
vIPO49	ACCTTGCTGCTCAAATGG	CTCCACAGTCGCCTCG
vIPO50	TAATGTGGGACCCAGTCG	CTCAAGCCCAACAAGAGC
vIPO52	CGCAAAGGCAGTTTTCC	GGATGGATTACATGACCGA
vIPO53	GCTCGGTGCTTCAAAGACC	TTGTTGATTTCTCCCTCATAC
vIPO54	GCACTTGTTTGCACGTCC	ACCCTCTCCAGCAACTCC
VIPO56	TGATCATACGACGAGACAG	CCCGTTCCCGAGAAGC
vanadium-dependent bromoperoxidase		
vBPO1	AAACTTCCACAAGACCCTC	GCACACACTCCAGCAGC
vBPO2	GCAATCACTGTAGAGCCTAA	TCTGGACTTCGGACACACC
vBPO3	CATCCCCAGGAGCAAGG	AGCATGAACTGAGACACAAC
vBPO4	TACCCCAGCGGTCACG	GAAACATCTCTGCCCCAGC
vBPO5	GCAGTTCCACAAGACACTCC	GCTCGTGAACACACACTCC
vBPO6	CGAACCCGTAATCTCGAACG	ATGCACTGACCCACAATCTC
vBPO7	GTCCAGAACGGTGCTTCC	GTCTCTGAACGCGATAGACG
vBPO8	GTTGTTTACCAGGGAAACA	GCAAGAACTGAGATACGAT
vBPO9	CCCAACCCCGTAGTCTCC	TTCTCATCCACGCACTCG
vBPO10	GTTACATCACCGCGCTTAAG	CTCCGTCGTTGGAGATTACG
vBPO11	TGTCACCCAACTGTTCAGG	GCTGTGCCACAATAGAGTCG
vBPO12	CAGTTCATGCTGTCAGACTT	GCCGTCATGTAGTCCAT
vBPO13	CAGCTCTGCAAGGGTGC	CGATTTCTTCAAAGGTCTCG
vBPO14	GTGCAGAACGGTGCTTCC	ATCCCTGAAGGCGATGC
vBPO15	ACGCTTCATCCGAAACG	ATGGAACCTCCAAGCACG
vBPO16	TCTCGCAGTTCGCCAGC	CTCGAAGTCCGACAAGAGG
vBPO17	GGAGACCACTGGCCTTA	GAACTCCGATGTGCGA

Supplementary Table 5. Sequences used for GDP-mannose 6-dehydrogenase phylogenetic analysis

Abbreviation	Accession Number
Aureococcus anophagefferens	XP_009034949.1
Arabidopsis thaliana	AAU90084.1
Blastopirellula marina	WP_002650323.1
*Chlamydomonas reinhardtii*01	BAF46285.1
*Chlamydomonas reinhardtii*02	XP_001698004.1
*Chlamydomonas reinhardtii*03	XP_001692910.1
Chondrus crispus	XP_005712525.1
*Coccomyxa subellipsoidea*01	XP_005646910.1
*Coccomyxa subellipsoidea*02	XP_005648704.1
*Cyanidioschyzon merolae*01	XP_005539438.1
*Cyanidioschyzon merolae*02	XP_005535140.1
Frankia sp. EuI1c	YP_004014117.1
Fibrobacter succinogenes	YP_003249048.1
Galdieria sulphuraria	XP_005707347.1
*Nannochloropsis gaditana*01	EWM24612.1
*Nannochloropsis gaditana*02	EWM24612.1
Paenibacillus curdlanolyticus	WP_006037536.1
Physcomitrella patens	EDQ52629.1
Phytophthora infestans	EEY67959.1
Prochlorococcus marinus	NP_895730.1
Pyropia yezoensis	contig_28523_g7013
*Thalassiosira pseudonana*01	XP_002293725.1
*Thalassiosira pseudonana*02	XP_002286770.1
*Volvox carteri*01	EFJ41942.1
*Volvox carteri*02	EFJ52903.1
*Volvox carteri*03	EFJ45696.1

Supplementary Table 6. Census of glycoside hydrolases (GH) and glycosyltransferases (GT) in *Saccharina japonica*

family	genes	family	genes	family	genes
GH1	1	GT1	3	GT47	14
GH2	3	GT2	24	GT48	2
GH3	1	GT4	13	GT49	1
GH5	2	GT7	1	GT50	1
GH10	1	GT8	5	GT57	2
GH13	1	GT10	2	GT58	1
GH16	3	GT13	1	GT60	1
GH17	2	GT14	1	GT64	3
GH18	1	GT20	4	GT68	2
GH30	1	GT22	2	GT74	1
GH37	1	GT23	17	GT76	1
GH47	2	GT25	1	GT77	11
GH81	53	GT27	1	GT90	1
GH85	1	GT28	4	GT92	2
GH88	1	GT31	4		
GH114	7	GT34	1		
GH128	1	GT41	4		
total	82	total	131		

Supplementary Table 7. Census of glycosyltransferases (GT) in *Saccharina japonica* and other algal genomes.

Familiy	Ectsi	Sacja	Auran	Phatr	Thaps	Chocr	Pyrye	Galsu	Nanoc	Nanog	Cocsu	Volca	Chlre	Cyame
**GT1**	1	3	2	1	0	0	1	0	1		1	0	5	0
**GT2**	10	24	7	5	4	3	9	4	3	4	1	2	4	0
**GT4**	13	13	11	7	6	5	6	9	6	6	2	2	6	3
**GT5**	0	0	0	0	0	1	1	1	0	0	2	4	8	0
**GT7**	1	1	1	1	0	4	0	0	0	0	0	0	0	0
**GT8**	3	5	6	8	4	3	1	5	2	2	0	1	1	1
**GT10**	1	2	2	3	0	0	0	1	1	1	1	0	1	0
**GT11**	0	0	0	0	0	0	0	2	0	0	0	0	0	1
**GT13**	2	1	1	1	1	2	1	2	0	0	0	0	0	0
**GT14**	5	1	0	2	1	9	6	5	2	0	0	0	0	0
**GT15**	1	0	0	1	3	0	0	0	2	0	0	0	0	0
**GT17**	0	0	0	0	0	0	0	1	0	0	2	0	0	0
**GT19**	0	0	0	0	0	0	0	4	0	0	0	0	0	1
**GT20**	6	4	5	2	3	4	4	6	3	3	0	1	3	2
**GT22**	3	2	0	3	3	1	0	1	1	2	2	0	0	0
**GT23**	7	17	0	0	0	0	3	0	1	1	1	1	1	0
**GT24**	1	0	2	1	0	1	2	1	1	1	1	1	1	0
**GT25**	3	1	9	1	1	0	2	3	0	0	0	0	0	0
**GT27**	0	1	2	0	1	2	0	0	0	0	0	0	0	0
**GT28**	0	4	3	4	3	5	1	4	0	1	0	2	2	1
**GT30**	0	0	0	0	0	0	0	1	0	0	0	0	1	0
**GT31**	0	4	0	4	1	0	0	4	1	0	4	1	0	0
**GT32**	0	0	0	6	0	0	0	0	0	0	0	0	1	0
**GT33**	1	0	1	1	1	1	0	1	1	1	1	1	1	1
**GT34**	1	1	0	1	1	1	0	1	1	2	0	2	2	0
**GT35**	0	0	0	0	0	1	1	3	0	0	2	2	2	1
**GT39**	0	0	0		0	6	5	2	0	0	0	0	0	2
**GT41**	3	4	3	1	1	1	0	1	0	0	0	0	1	0
**GT45**	0	0	0	0	0	1	1	0	0	0	0	0	0	0
**GT47**	8	14	7	4	1	3	2	1	0	0	2	26	38	0

**GT48**	3	2	2	1	1	0	0	0	1	1	0	4	7	0
**GT49**	3	1	2	6	2	0	0	1	1	2	0	8	4	1
**GT50**	1	1	1	1	1	0	0	0	0	1	0	1	0	0
**GT54**	1	0	0	0	0	0	0	0	0	0	0	0	0	0
**GT57**	2	2	1	2	2	2	0	2	1	1	0	2	2	1
**GT58**	1	1	1	1	1	1	0	1	0	0	0	0	0	0
**GT59**	1	0	0	0	0	2	0	1	1	1	1	1	1	0
**GT60**	3	1	4	5	8	0	0	0	5	4	2	1	3	0
**GT62**	0	0	2	0	0	0	0	0	0	0	0	0	0	0
**GT64**	2	3	0	3	2	2	0	3	2	0	0	0	1	0
**GT65**	1	0	0	0	0	0	0	0	0	0	0	0	0	0
**GT66**	1	0	0	2	2	1	1	0	0	0	1	2	2	1
**GT68**	0	2	1	2	6	0	0	0	0	1	0	0	0	0
**GT69**	0	0	1	0	0	0	0	5	2	1	0	2	0	1
**GT71**	0	0	0	0	0	0	0	0	4	1	2	1	4	2
**GT74**	1	1	0	0	1	0	0	0	0	0	0	0	0	0
**GT75**	0	0	3	0	0	0	0	0	0	0	1	3	1	0
**GT76**	1	1	0	0	0	0	0	0	0	0	0	0	1	0
**GT77**	1	11	6	7	5	5	4	2	3	1	2	13	16	2
**GT78**	0	0	0	0	0	1	0	0	2	0	0	0	0	0
**GT83**	0	0	0	0	0	0	0	1	0	0	0	0	0	0
**GT90**	0	1	0	10	0	2	1	5	2	0	1	10	31	1
**GT92**	0	2	0	0	0	1	0	1	0	0	1	0	0	0
**total**	**92**	**131**	**86**	**97**	**66**	**71**	**52**	**85**	**50**	**38**	**33**	**94**	**151**	**22**

Blue boxes stand for the lack families in *Saccharina* compared to *Ectocarpus* and red boxes stand for the extra families in *Saccharina* compared to *Ectocarpus*. *Nanoc=Nannochloropsis oceanica*， *Nanga= Nannochloropsis gaditana*, *Auran= Aureococcus anophagefferens*, Ectsi= *Ectocarpus siliculosus*, *Sacja= Saccharina japonica*, *Thaps= Thalassiosira pseudonana*, *Phatr= Phaeodactylum tricornutum*, *Chocr=Chondrus crispus*, *Cyame= Cyanidioschyzon merolae* , *Pyrye=Pyropia yezoensis*, *Galsu=Galdieria sulphuraria*, *Chlre= Chlamydomonas reinhardtii*, *Cocsu=Coccomyxa subellipsoidea*, *Volca=Volvox carteri.*

Supplementary Table 8. Census of glycoside hydrolases (GH) in *Saccharina japonica* and other algal genomes.

Familiy	Ectsi	Sacja	Auran	Phatr	Thaps	Chocr	Pyrye	Gals	Nanoc	Nanog	Cocsu	Volca	Chlre	Cyame
**GH1**	3	1	1	1	1	0	1	0	3	3	5	3	2	0
**GH2**	2	3	4	0	1	2	0	1	1	1	3	0	1	0
**GH3**	1	1	8	3	0	0	0	0	4	4	0	0	0	0
**GH5**	2	2	3	5	1	1	2	0	8	8	1	1	4	0
**GH6**	0	0	0	0	0	2	0	0	0	0	0	0	0	0
**GH8**	0	0	0	0	0	0	0	0	3	2	2	0	0	0
**GH9**	0	0	0	0	1	0	0	0	5	5	3	3	3	0
**GH10**	1	1	0	1	0	0	0	0	0	0	0	2	0	0
**GH13**	0	1	0	0	0	5	4	9	0	0	3	7	11	6
**GH14**	0	0	0	0	0	1	1	5	0	0	0	2	3	1
**GH15**	0	0	0	0	0	0	0	3	0	0	0	0	0	0
**GH16**	6	3	9	6	5	3	2	0	1	1	1	2	4	0
**GH17**	1	2	0	0	0	0	0	0	0	1	0	0	0	0
**GH18**	0	1	4	2	16	0	1	0	0	1	0	1	12	0
**GH19**	0	0	0	0	7	0	0	0	0	0	0	0	0	0
**GH20**	0	0	5	2	0	0	0	0	0	0	0	0	0	0
**GH27**	0	0	1	0	0	0	0	1	0	0	1	0	0	0
**GH28**	0	0	8	3	0	0	0	0	0	0	0	0	1	0
**GH29**	0	0	1	3	0	0	0	0	0	0	0	0	0	0
**GH30**	1	1	1	1	1	0	0	2	0	0	1	0	0	0
**GH31**	1	0	4	1	2	2	2	6	2	2	4	0	2	5
**GH32**	0	0	4	0	0	0	0	0	0	0	0	3	4	0
**GH33**	0	0	0	0	0	0	0	0	0	0	1	0	0	0
**GH35**	0	0	2	1	0	2	2	7	0	0	2	0	0	1
**GH36**	2	0		0	0	1	3	3	1	0	0	0	0	1
**GH37**	1	1	1	0	0	1	0	1	2	2	0	1	1	2
**GH38**	1	0	1	2	1	1	0	2	1	1	0	1	1	0
**GH39**	0	0	1	0	0	0	0	0	0	0	0	0	0	0
**GH43**	0	0	2	0	1	0	0	0	0	1	0	0	0	0

**GH45**	0	0	3	0	0	1	0	0	0	0	0	0	0	0
**GH47**	5	2	5	1	4	4	1	1	2	3	4	1	0	1
**GH54**	0	0	5	0	0	0	0	0	0	0	0	0	0	0
**GH63**	1	0	0	0	0	1	0	0	0	0	0	0	0	0
**GH65**	0	0	0	0	0	0	0	0	1	0	0	0	0	0
**GH67**	0	0	2	0	0	0	0	0	0	0	0	0	0	0
**GH72**	0	0	0	1	0	0	0	0	0	0	0	0	0	0
**GH76**	0	0	1	0	0	0	0	0	0	0	0	0	0	0
**GH77**	0	0	0	0	0	1	1	1	0	0	0	2	2	2
**GH78**	0	0	3	0	4	0	0	0	1	0	0	0	0	0
**GH79**	0	0	2	0	0	0	0	0	1	0	0	0	0	0
**GH81**	18	53	1	0	0	0	0	0	1	1	0	1	3	0
**GH85**	1	1	0	0	0	1	1	1	0	1	0	0	1	1
**GH88**	6	1	0	0	0	0	0	0	0	0	0	0	0	0
**GH89**	0	0	0	0	0	0	0	0	0	0	1	0	0	0
**GH92**	0	0	2	0	0	0	0	0	0	0	0	0	0	0
**GH95**	1	0	2	0	0	0	0	0	0	0	0	0	0	0
**GH97**	0	0	0	0	0	0	1	0	0	0	0	0	0	0
**GH99**	0	0	2	1	0	0	0	0	0	0	0	1	1	0
**GH105**	0	0	2	0	0	0	0	0	0	0	0	0	0	0
**GH109**	0	0	0	1	4	0	4	0	0	0	0	0	0	1
**GH113**	0	0	0	0	0	0	4	0	0	0	0	0	0	0
**GH114**	0	7	0	0	0	0	0	0	0	0	0	1	2	0
**GH125**	0	0	0	2	1	0	0	0	0	0	0	1	1	0
**GH128**	0	1	0	0	0	0	0	0	0	0	1	0	0	0
**total**	**54**	**82**	**90**	**37**	**50**	**31**	**30**	**43**	**37**	**37**	**33**	**33**	**59**	**21**

Blue boxes stand for the lack families in *Saccharina* compared to *Ectocarpus* and red boxes stand for the extra families in *Saccharina* compared to *Ectocarpus*. *Nanoc=Nannochloropsis oceanica*，*Nanga= Nannochloropsis gaditana*, *Auran= Aureococcus anophagefferens*, Ectsi= *Ectocarpus siliculosus*, *Sacja= Saccharina japonica*, *Thaps= Thalassiosira pseudonana*, *Phatr= Phaeodactylum tricornutum*, *Chocr=Chondrus crispus*, *Cyame= Cyanidioschyzon merolae* , *Pyrye=Pyropia yezoensis*, *Galsu=Galdieria sulphuraria*, *Chlre= Chlamydomonas reinhardtii*, *Cocsu=Coccomyxa subellipsoidea*, *Volca=Volvox carteri.*

Supplementary Notes

Supplementary Note 1. Carbon storage and cell wall metabolism

The analogous set of genes involved in the polysaccharide biosynthesis metabolism pathways for mannitol, trehalose, cellulose, laminarin, alginate, sulfate fucan and sucrose in the 14 algal genomes were identified and annotated based on KEGG and previous functional classifications[1, 2, 3, 4] (**Supplementary Figure 12**).

Mannitol is one of the most widespread sugar alcohol compounds widely found in bacteria, fungi, algae, and land plants[1]. It is known to be involved in osmoregulation, the storage and regeneration of reducing power, and serves as a compatible solute in both land plants and algae[2]. Genes involved in the mannitol cycle in *Ectocarpus* were obtained from a previous study[3] as reference genes to search against *S. japonica* and the other algae genomes using BLASTp. A cutoff E-value of $<10^{-10}$ was set to pare BLAST results to obtain candidate genes. All of the identified proteins were manually curated by searching against the NCBI non-redundant protein database. As shown in **Supplementary Figure 12**, the complete mannitol cycle was only identified in the stramenopile algae *Saccharina, Ectocarpus* and *Nannochloropsis.* One ortholog of M1PHD (55.7% identity to bacteria) and two orthologs of M1pase (>42.0% identity to bacteria), which facilitate mannitol biosynthesis, were identified in the *S. japonica* genome. One ortholog of M2HD (55.6% identity to bacteria) and one ortholog of FK (50.3% identity to bacteria), which facilitate mannitol hydrolysis, were identified in the *S. japonica* genome. Phylogenetic trees supported the HGT origin of the mannitol cycle in brown seaweeds (**Supplementary Figure 13**).

Laminarin is another carbon storage compound in brown algae[4]. Unlike land plants, brown algae do not store the carbon assimilated by photosynthesis as insoluble starch granules but instead as the soluble 1,3-β-glucan polymer (laminarin) localized to the cytosol[5]. Genes involved in the biosynthesis of laminarin were compared among all available algal genomes. Our results showed that laminarin could be synthesized in stramenopiles and green algae but not in red algae because the genes needed for the final two steps, a β-1,3-glucan synthase gene from the GT48 family and a KRE6-like gene belonging to the GH16 family, were not found in red algae. These two genes are likely involved in the synthesis of the β- 1,6-linked branches of laminarin[4].

Alginate is a major cell wall polymer of brown algae, accounting for up to 45% of the dry weight. It is an unbranched polysaccharide initially synthesized as a β-1,4-D-mannuronic acid chain (M-alginate). The precursor for M-alginate is GDP-mannuronic acid, which is believed to be derived from a four-electron oxidation of GDP-mannose by the enzyme GMD[6]. The M-alginate is later modified by MC5Es, which convert single residues or large blocks of polymers from D-mannuronic acid into L-guluronic acid (G-alginate). The MC5E genes have been reported in the brown algae *Laminaria digitata* and *E. siliculosus*. Among all 14 algal genomes in our study, MC5Es were only found in *Ectocarpus* and *S. japonica*, indicating that other stramenopiles as well as red algae and green algae should not be able to synthesize alginate. The identification of 105 MC5E genes in *S. japonica* has exceeded the

record of 28 of these genes identified in *E. siliculosus*. Genetic distance analysis of MC5Es showed that 43 of these genes were on seven scaffolds and shared high sequence similarity (>85%), indicating that recent tandem duplication events occurred in MC5E evolution. Phylogenetic analysis of MC5Es and GDPs among the genomes of *Ectocarpus, S. japonica* and other algae suggests that putative HGT events from Actinobacteria introduced the chimeric pathway to brown algae.

Sulfated fucans are matrix polysaccharides from the cell wall of marine fucalean brown algae, consisting of an α-L-fucose backbone substituted by sulfate ester groups and branched with other monosaccharide residues[7]. In sulfated fucan metabolism, the *E. siliculosus* genome possesses two candidate pathways to metabolize GDP-fucose, which is the fucan precursor. One pathway is catalyzed by GDP-mannose 4,6-dehydratase and GDP-L-fucose synthetase, and the other is an alternative salvage pathway that is catalyzed by L-fucokinase (FK) and GDP-fucose pyrophosphorylase. Fucosyltransferases (FTs) from the glycosyltransferase (GT) families (e.g., GT10, GT23, and GT65) can be involved in the polymerization of GDP-fucose into elongating fucan chains in *E. siliculosus*, and polymerized polysaccharides are sulfated by specific sulfotransferases. Based on the known sulfated fucan pathway, we reconstructed the metabolic pathway for the biosynthesis and remodeling of sulfated fucans in *S. japonica* and the other 13 algae genomes. Red algae lack candidate genes in two pathways to metabolize GDP-fucose, indicating the absence of sulfated fucans or the existence of an alternative pathway. By comparing the genes involved in the last two steps, FTs (6 in *S. japonica*, 5 in *E. siliculosus*, and more than 3 in other algae) and sulfotransferase (24 in *S. japonica*, 23 in *E. siliculosus*, and more than 10 in other algae), brown algae showed an obvious advantage in gene numbers, consistent with the sophisticated structure of their cell wall matrix.

Supplementary Note 2. Carbohydrate-active enzymes

Carbohydrate-active enzymes (CAZymes) are responsible for the breakdown, biosynthesis or modification of glycoconjugates, oligo- and polysaccharides[8]. CAZymes can be subdivided into four functional classes based on their structurally related catalytic modules or functional domains: glycoside hydrolases (GHs), glycosyltransferases (GTs), polysaccharide lyases (PLs), and carbohydrate esterases (CEs). Among them, the key enzymes for the synthesis and remodeling of oligo- and polysaccharides are GHs and GTs, which are classified into more than 200 Carbohydrate-Active enZYme (CAZY) families (http://www.cazy.org/)[9]. To identify the CAZymes from *S. japonica* and distinguish the different cell wall polysaccharides in other algae, we performed CAZyme screening in *S. japonica* and the other 13 algal genomes (**Supplementary Tables 6-8**). All of the putative proteins were searched against entries in the CAZy database using the dbCAN Web server[10], in which HMMer[11] was used to query against a collection of custom-made HMM profiles constructed for each CAZy family. The original output was downloaded and parsed manually with the following parameters: 1) E-value $<10^{-10}$; 2) identity >50%; and 3) alignment length >80 amino acids. All of the identified proteins were then manually curated.

A total of 213 putative CAZymes were identified in *S. japonica* (**Supplementary Table 6**) using the CAZy annotation pipeline. The genome of *S. japonica* encodes 82 genes from 17 GH families and 131 genes from 30 GT families, which represents a higher absolute number of genes than in *Ectocarpus* but fewer gene families (54

genes from 18 GH families and 92 genes from 32 families of GT). Gene expansion of the GT families was found in GT2, GT23, GT47 and GT77 containing 24, 17, 14 and 11 genes, respectively, in *S. japonica,* compared with 12, 7, 8 and 1 genes, respectively, in *Ectocarpus*. These four families are related to cellulose and alginate biosynthesis in brown algae, providing additional evidence for the morphological enhancement of *S. japonica*. Furthermore, compared with *Ectocarpus*, *S. japonica* gains several new GT families, namely GT27, GT28, GT31, GT68, GT90, and GT92, which are described as acetylglucosaminyltransferases or O-α-fucosyltransferases, but lacks other GT families, such as GT15, GT24, GT33, GT54, GT59, GT65, GT66, which each contain a single gene in each family in *S. japonica*.

Compared with the GH families in *Ectocarpus*, 4 (GH13, GH18, GH114, GH128) were newly gained and an additional 4 (GH31, GH36, GH63, GH95) were lost in *S. japonica*. Among them, 7 genes in GH114 were found exclusively in *S. japonica*. The gene expansion of GH families was found in GH81 (mainly including endo-1,3-β-glucanases). The comparison of gene numbers and families within the GH and GT family groups between the two brown algae provides additional insight into the evolution and diversification of cell wall related polysaccharides in *S. japonica*.

Supplementary Methods

Sample collection and gametophytes separation

The female gametophytes of 12 *S. japonica* species used for DNA and RNA sequencing were separated and maintained in the Yellow Sea Fisheries Research Institute Algae Culture Center, Qingdao, China. Methods for gametophyte separation are described as follows: Mature sporophylls with sporangia were collected and cleaned of epibionts and rinsed with filtered seawater. Then, the sorus was excised and maintained in darkness on moist paper towels at 10°C for 1–2 h. Zoospores were subsequently released from the sorus as soon as it was re-immersed in seawater. They were then cultured aseptically in PES medium at 10±1°C and 20 μmol photons m^{-2} s^{-1} with a 14:10 h light/dark photoperiod for two weeks. Then, male and female gametophytes were distinguished under a microscope according to size and were preserved separately at 10±1°C and 5 μmol photons m^{-2} s^{-1} with a 12:12 h light/dark photoperiod. Mature fronds of *Saccharina* sp. (B, E, F, G) were collected from the Pacific coast of the Russian Far East (**Supplementary Table 1**), and sporophytes were cultivated via gametophyte cloning technology in Rongcheng, China. Mature fronds of the late-maturing *S. japonica* strain W were sampled from Rongcheng, China.

DNA isolation

The DNA extraction protocol was based on the method described by Apt et al. [12] and was optimized for *Saccharina*. A fresh sample was ground in liquid nitrogen and then incubated in extraction buffer (100 mM Tris-HCl, pH 7.5, 2 M NaCl, 2% CTAB, 50 mM EDTA, pH 7.5, and 50 mM DTT) at 55°C for 2 hours in the presence of RNaseA. After centrifugation for 10 min at 10,000×g, the supernatant was transferred to a new tube, and one-third volume of absolute ethanol and one-ninth volume of 3 M potassium acetate (pH 4.8) was added slowly and mixed by gentle agitation. The mixture was extracted with one volume of phenol-chloroform (1:1, v/v), and then with one volume of chloroform:isoamyl alcohol (24:1, v/v). The DNA was precipitated from the supernatant by the addition of 0.8 volumes of isopropanol,

incubated at 4°C for 30 min, and then collected by centrifugation at 13,000×g for 30 min. The DNA pellet was washed with ice-cold 70% ethanol and then dissolved in an appropriate volume of nuclease-free deionized water (pH 8.0).

RNA isolation

The isolation of high-quality RNA from brown seaweed has always been problematic because of the high content of polyphenolic compounds and polysaccharides in their tissues. The protocol used for RNA extraction was based on the method developed by Bail et al.[13] with some modifications. Fresh tissue was ground in liquid nitrogen and immediately incubated in the presence of extraction buffer (100 mM Tris-HCl, pH 8.0, 2 M NaCl, 20 mM EDTA, and 2% CTAB [w/v]), and then 50 mM DTT was added before extraction at a ratio of 1:10 [tissue (w)/buffer (v)]. The mixture was mixed vigorously, incubated at room temperature for 30 min, and then centrifuged for 10 min at 10,000×g. The supernatant was transferred to a new tube, and one-third volume of absolute ethanol and one-ninth volume of 3 M potassium acetate (pH 4.8) was added slowly and mixed by gentle agitation. The mixture was extracted with one volume of chloroform:isoamyl alcohol (24:1, v/v, pH 8.0) and then centrifuged at 12,000×g for 20 min. The supernatant was transferred to a new tube, and RNA was precipitated by the addition of 0.25 volumes of 12 M LiCl followed by overnight incubation at -20°C. The RNA pellet was collected by centrifugation at 14,000×g for 30 min at 4°C. DNase treatment was conducted using RNase-free DNase I (Promega) according to the manufacturer's instructions to eliminate any residual genomic DNA from the preparation. An extraction was then performed by adding phenol-chloroform (1:1, v/v). Following centrifugation, the upper phase was transferred to a fresh tube and extracted with one volume of chloroform:isoamyl alcohol (24:1, v/v). The upper phase was precipitated with 0.3 M NaAc (pH 5.5) and 75% ice-cold ethanol by incubating overnight at -20°C. The RNA pellet was recovered by centrifugation and then washed with ice-cold 70% ethanol and air-dried. Finally, the RNA pellet was dissolved in an appropriate volume of DEPC-treated water.

Determination of Iodine contents in *S. japonica* sporophytes

Brown algae of Laminariaceae are generally thought to be the strongest iodine accumulators among living organisms, with an average content of 1.0% (up to 5%) of dry weight. Iodine provides these algae an abundant and accessible source for potential chemical defense and antioxidative activity[14].

The accumulated iodine content in adult *S. japonica* sporophytes was assayed by two methods. In the first method, the amount of iodine anion in a dried sample was determined by iodometry after the sample had been burnt to ash. The iodine contents of the basal blades, middle blades and distal blades were 0.26%, 0.66%, 1.34% of dry weight, respectively (**Supplementary Figure 11**). In the second method, the amount of iodine was determined by scanning electron microscopy (SEM) analyses. Sporophytes of *S. japonica* (approximately 1 m in length) were rapidly washed in deionized water. Tissue samples were taken from the holdfast, stipe, basal blades, middle blades, and distal blades. Cross-sections and surface parts were cut using a scalpel, immediately cryofixed in liquid nitrogen-chilled isopentane and freeze-dried in a cryostat at -50°C for 5 h[15]. Ten elements [oxygen (O), sodium (Na), magnesium (Mg), sulfur (S), chlorine (Cl), potassium (K), calcium (Ca), bromine (Br), rhodium (Rh), antimony (Sb), and iodine (I)] were determined by X-ray analysis using SEM

with energy-dispersive spectrometry. The energy levels of the peaks in these spectra provide qualitative data on the elements existence in the sample. The peak amplitudes also provide quantitative data in terms of the percentage concentration of each element. The percentage concentration of iodine was determined at four parts: holdfast and stipe (0.05%), basal blades (0.03%), middle blades (0.06%) and distal blades (0.35%) (**Supplementary Figure 12**). Overall, the variation tendency in the iodine contents in different parts of adult sporophytes was similar between the two methods, and distal blades contain much more iodine than other parts. These results were similar to the results in *L. digitata*. Additionally, plantlets of *L. digitata* (less than 15 cm in length) were very rich in iodine compared with adults[16].

Expression analysis of identified vHPO genes in *S. japonica*

Transcriptional regulation of vHPO was shown to be efficient for switching to specialized iodine metabolism for antioxidative capacities[17]. The expression of vBPO and vIPO genes was investigated by real-time PCR in gametophytes, juvenile sporophytes and different tissues of adult sporophytes, including holdfast, stipe, basal blades, middle blades, and distal blades. From each RNA sample, 1 μg of RNA was reverse transcribed to cDNA using oligo(dT)18 and the Superscript™ First-Strand synthesis for RT-PCR (Invitrogen) according to the manufacturer's instructions. Primers were designed by using Primer Express TM1.0 (PE Applied Biosystems, Foster City, CA, USA) (**Supplementary Table 4**). The real-time PCR reactions were performed in a 96-well thermocycler (ABI StepOne Plus) with SYBR green reaction mix from TAKARA for 3 min at 95°C, followed by 40 runs of 10 sec at 95°C and 1 min at 60°C. Each sample was technically duplicated. The specificity of amplification was checked with a dissociation curve obtained by heating the samples from 65°C to 95°C. Triplicate qPCRs were performed for each sample. The $2^{-\Delta\Delta CT}$ method[18] was used to analyze the quantitative real-time PCR data. Average-linkage hierarchical clustering and heat maps were generated in R Bioconductor using the heatmap.2 function (omitting row and column dendrograms) in the gplots package of the R program.

Phylogenetic analysis of GMDs and MC5Es

Homologous protein sequences were aligned with MUSCLE[7]. Poorly aligned regions were trimmed before the sequences were realigned. The process was repeated until no further improvements could be made. Positions with gaps were removed before construction of phylogenies. ProtTest[20] was used to select the best model of protein evolution for each set of proteins. Maximum-likelihood trees were constructed using PhyML v3.0[21] under the following parameters: 100 bootstrap replicates; a four-category gamma distribution; and the proportion of the variable sites was estimated from the data.

Identification of candidate selective genes

Homozygous SNVs from 9 wild individuals and 8 cultivars were used to identify candidate selective regions. All of the assembled scaffolds were first concatenated and then divided into 200-kb bins. The average number of pairwise nucleotide differences, the average number of segregating sites and Tajima's D were calculated for each bin. Bins with Z-test scores of Tajima's D lower or higher than 5% of all bins were identified as candidate selective regions. Protein-coding genes in these selective regions were considered candidate selective genes. Thus, four selective gene groups

(cultivar-neg, cultivar-pos, wild-neg, wild-pos) were generated, which contained 828, 674, 659, and 566 genes, respectively (**Supplementary Datas 4-7**). Gene Ontology-based annotation of these genes was used for gene set enrichment analysis. TopGO was used to detect Gene Ontology terms and gene families enriched in each of the four groups using a hypergeometric test with Benjamini-Hochberg correction (FDR cutoff of 0.10) with the *S. japonica* JA annotation as the background.

Supplementary References

1. Iwamoto K, Shiraiwa Y. Salt-regulated mannitol metabolism in algae. *Marine biotechnology* **7**, 407-415 (2005).
2. Iwamoto K, Kawanobe H, Ikawa T, Shiraiwa Y. Characterization of salt-regulated mannitol-1-phosphate dehydrogenase in the red alga Caloglossa continua. *Plant physiology* **133**, 893-900 (2003).
3. Michel G, Tonon T, Scornet D, Cock JM, Kloareg B. Central and storage carbon metabolism of the brown alga Ectocarpus siliculosus: insights into the origin and evolution of storage carbohydrates in Eukaryotes. *The New phytologist* **188**, 67-81 (2010).
4. Cock JM, *et al.* The Ectocarpus genome and the independent evolution of multicellularity in brown algae. *Nature* **465**, 617-621 (2010).
5. Charrier B, *et al.* Development and physiology of the brown alga Ectocarpus siliculosus: two centuries of research. *The New phytologist* **177**, 319-332 (2008).
6. Tenhaken R, Voglas E, Cock JM, Neu V, Huber CG. Characterization of GDP-mannose dehydrogenase from the brown alga Ectocarpus siliculosus providing the precursor for the alginate polymer. *The Journal of biological chemistry* **286**, 16707-16715 (2011).
7. Barbeyron T, L'Haridon S, Michel G, Czjzek M. Mariniflexile fucanivorans sp. nov., a marine member of the Flavobacteriaceae that degrades sulphated fucans from brown algae. *International journal of systematic and evolutionary microbiology* **58**, 2107-2113 (2008).
8. Zhao Z, Liu H, Wang C, Xu JR. Comparative analysis of fungal genomes reveals different plant cell wall degrading capacity in fungi. *BMC genomics* **14**, 274 (2013).
9. Cantarel BL, Coutinho PM, Rancurel C, Bernard T, Lombard V, Henrissat B. The Carbohydrate-Active EnZymes database (CAZy): an expert resource for Glycogenomics. *Nucleic acids research* **37**, D233-238 (2009).
10. Yin Y, Mao X, Yang J, Chen X, Mao F, Xu Y. dbCAN: a web resource for automated carbohydrate-active enzyme annotation. *Nucleic acids research* **40**, W445-451 (2012).
11. Eddy SR. A new generation of homology search tools based on probabilistic inference. *Genome informatics International Conference on Genome Informatics* **23**, 205-211 (2009).
12. Apt KE, Clendennen SK, Powers DA, Grossman AR. The gene family encoding the fucoxanthin chlorophyll proteins from the brown alga Macrocystis pyrifera.

Molecular & general genetics : MGG **246**, 455-464 (1995).
13. Le Bail A*, et al.* Normalisation genes for expression analyses in the brown alga model Ectocarpus siliculosus. *BMC molecular biology* **9**, 75 (2008).
14. Kupper FC*, et al.* Commemorating two centuries of iodine research: an interdisciplinary overview of current research. *Angewandte Chemie* **50**, 11598-11620 (2011).
15. Verhaeghe EF*, et al.* Microchemical imaging of iodine distribution in the brown alga Laminaria digitata suggests a new mechanism for its accumulation. *Journal of biological inorganic chemistry : JBIC : a publication of the Society of Biological Inorganic Chemistry* **13**, 257-269 (2008).
16. Küpper FC, Schweigert N, Ar Gall E, Legendre JM, Vilter H, Kloareg B. Iodine uptake in Laminariales involves extracellular, haloperoxidase-mediated oxidation of iodide. *Planta* **207**, 163-171 (1998).
17. Cosse A, Potin P, Leblanc C. Patterns of gene expression induced by oligoguluronates reveal conserved and environment-specific molecular defense responses in the brown alga Laminaria digitata. *The New phytologist* **182**, 239-250 (2009).
18. Livak KJ, Schmittgen TD. Analysis of relative gene expression data using real-time quantitative PCR and the 2(-Delta Delta C(T)) Method. *Methods* **25**, 402-408 (2001).
19. Edgar RC. MUSCLE: multiple sequence alignment with high accuracy and high throughput. *Nucleic acids research* **32**, 1792-1797 (2004).
20. Abascal F, Zardoya R, Posada D. ProtTest: selection of best-fit models of protein evolution. *Bioinformatics* **21**, 2104-2105 (2005).
21. Guindon S, Delsuc F, Dufayard J-F, Gascuel O. Estimating maximum likelihood phylogenies with PhyML. In: *Bioinformatics for DNA Sequence Analysis* (ed^(eds). Springer (2009).
